# A RUBY-Based Visual Hairy Root Transformation and CRISPR-*PTG* Genome-Editing System in *Eucommia ulmoides*

**DOI:** 10.3390/plants15182795

**Published:** 2026-09-11

**Authors:** Linqing Xu, Yani Zhou, Yongfeng Sun, Yating Dong, Rong Kang, Nan Yao, Xia Cai

**Affiliations:** Key Laboratory of Resource Biology and Biotechnology in Western China, Ministry of Education, Northwest University, Xi’an 710069, China; 202021135@stumail.nwu.edu.cn (L.X.); zhouyani@163.com (Y.Z.); sunyongfeng@stumail.nwu.edu.cn (Y.S.); 17835847242@163.com (Y.D.); 18891464558@163.com (R.K.); nanyao@stumail.nwu.edu.cn (N.Y.)

**Keywords:** *Agrobacterium*, hairy root transformation, RUBY, CRISPR-PTG, *EuSQS*, *Eucommia ulmoides*

## Abstract

*Eucommia ulmoides* Oliv. is a woody plant with significant medicinal and industrial value. However, the lack of an efficient genetic transformation and gene-editing system has seriously hindered the research on its functional genes and the progress of molecular breeding. To break through this technical bottleneck, this study successfully established an efficient *Agrobacterium*-mediated genetic transformation system for *E. ulmoides* hairy roots based on the RUBY visual reporter gene. Through systematic optimization, the optimal transformation conditions were identified as infecting hypocotyl explants with *Agrobacterium* strain K599 at an OD_600_ of 0.6 for 20 min. Based on this optimized system, a RUBY-based CRISPR-PTG (polycistronic tRNA-gRNA) construct was further generated. With squalene synthase (*EuSQS*) as the target gene, Sanger sequencing and ICE (Inference of CRISPR Edits) analysis provided preliminary evidence for CRISPR-PTG-mediated editing at the *EuSQS* locus in transgenic hairy roots, although the potentially chimeric nature of hairy roots may contribute to variation in the observed editing profiles. In this study, a RUBY-based visual hairy root genetic transformation system was established, which provided preliminary evidence that CRISPR-PTG-mediated targeted mutagenesis can occur in *E. ulmoides* hairy roots. These results provide a useful basis for further optimization and application of genome editing in subsequent gene function analysis and metabolic engineering research of *E. ulmoides*, and also offer a reference for genetic transformation studies of other woody plants.

## 1. Introduction

*Eucommia ulmoides*, as a perennial woody plant, has significant application value in both traditional medicine and industrial production [1,2]. Its tissues accumulate diverse secondary metabolites such as terpenoids, flavonoids, and polysaccharides. These compounds play important roles in lowering blood pressure and reducing inflammation, making *E. ulmoides* widely used in medical research [3,4]. Moreover, the whole plant of *E. ulmoides* contains Eucommia rubber, which serves as an important source of natural rubber [5]. Progress in genomic and molecular biological approaches has uncovered numerous functional genes governing secondary metabolism as well as growth and developmental processes in *E. ulmoides* [6]. However, the absence of reliable and high-efficiency tissue culture and genetic transformation system for this species has severely restricted the progress of its functional gene research and molecular breeding. Currently, the *Agrobacterium*-mediated hairy root transformation system has been extensively applied in woody plants owing to its high efficiency, stable genetic background and short time period [7,8]. This system also provides crucial technical support for functional analysis of target genes. Therefore, establishing a highly effective *Agrobacterium*-mediated hairy root genetic transformation system for *E. ulmoides* is a critical technical bottleneck that urgently needs to be overcome.

Most studies have reported that the selection of transgenic plants often relies on reporter genes, including fluorescent protein genes such as GFP and RFP, as well as enzymatic reporter genes represented by β-glucuronidase (GUS) [9,10]. Although the incorporation of these fusion reporter genes enables the selection of transgenic plant materials, it often requires exogenous substrates, expensive instruments or destructive detection. These requirements severely restrict both the efficiency and the number of positive transgenic materials, thereby constraining subsequent functional studies. At present, the RUBY reporter system which was developed based on the betalain biosynthesis pathway [11] catalyzes the conversion of tyrosine into bright red betalains in plants through a series of enzymatic reactions involving *CYP76AD1*, *DODA* and glucosyl transferase. This allows rapid, non-destructive visual screening of transgenic plant materials, greatly simplifying the screening procedure after plant transformation [12]. Although a RUBY-based genetic transformation system has been established in several woody plant species [13,14], the research using the RUBY reporter in *E. ulmoides* has not been reported yet. Therefore, constructing a RUBY-based visual genetic transformation system for hairy roots in *E. ulmoides* is of great significance for elucidating gene functions in this species.

CRISPR-Cas9, a dominant plant genome editing technology, has become a primary tool in this field [15]. The targeting precision and editing efficiency of this system in plant genomes are determined by the gRNAs. On this basis, the current study developed a novel editing strategy that utilizes the endogenous tRNA processing system to synthesize a polycistronic tRNA-gRNA (PTG) construct, thereby enabling the production of multiple gRNAs through a single transcript in plants. In this construct, the gRNA sequences are separated by tRNA spacers, which are recognized and cleaved by the endogenous tRNA-processing system, resulting in the release of individual functional gRNAs [16,17]. Moreover, multiple gRNAs can simultaneously target multiple regions of the same gene, thereby enhancing the editing efficiency of CRISPR-Cas9 in plant genomes [18]. The CRISPR-PTG strategy has recently been applied in several woody plants. In *grapevine*, the use of a PTG expression cassette significantly enhanced editing efficiency [19], and a tRNA-based sgRNA array significantly enhanced the efficiency of multiplex gene editing in *Citrus* [20]. Therefore, the development of a CRISPR-PTG-based method could provide new guidance for functional studies of key genes involved in secondary metabolism of *E. ulmoides*.

Among genes associated with terpenoid metabolism, squalene synthase (*SQS*) catalyzes a rate-limiting step in the plant terpenoid biosynthesis pathway and participates in plant growth, development and environmental stress adaptation [21,22]. For instance, overexpression of *AtSQS* promotes overall growth and reproductive development, while overexpression of *TgSQS* in *Arabidopsis* enhances drought tolerance [21,23]. Furthermore, in medicinal plants, the function of *SQS* is closely related to the biosynthesis of their bioactive components [24]. In *Withania somnifera*, silencing *WsSQS* significantly decreased the content of its major bioactive withanolides [25], whereas overexpression of *PgSQS* has been shown to promote the accumulation of triterpenoid saponins [26]. Collectively, SQS plays important roles in plant growth and development, stress responses, and terpenoid secondary metabolism. However, the biological functions of *EuSQS* in *E. ulmoides* remain unclear. Therefore, *EuSQS*, as an endogenous gene with potential relevance to terpenoid metabolism and to the medicinal value of *E. ulmoides*, was selected to assess the feasibility of CRISPR-PTG-mediated genome editing in this species.

In this study, a genetic transformation system for *E. ulmoides* hairy roots based on the RUBY reporter gene was established. By systematically optimizing key parameters such as the type of explants, *Agrobacterium* strains, bacterial concentration, and infection time, we established a complete and standardized genetic transformation protocol for *E. ulmoides* hairy roots. Furthermore, we developed a new CRISPR-PTG-RUBY system that can be used for rapid screening by integrating the RUBY reporter gene. We used *EuSQS* as the target gene and evaluated the feasibility of this system in the genome editing of *E. ulmoides* using Sanger sequencing and ICE analysis. This work provides a preliminary basis for future gene function studies and molecular breeding in this important medicinal woody plant.

## 2. Results

### 2.1. Construction of a RUBY-Based Visualization System in E. ulmoides

**Figure 1:**
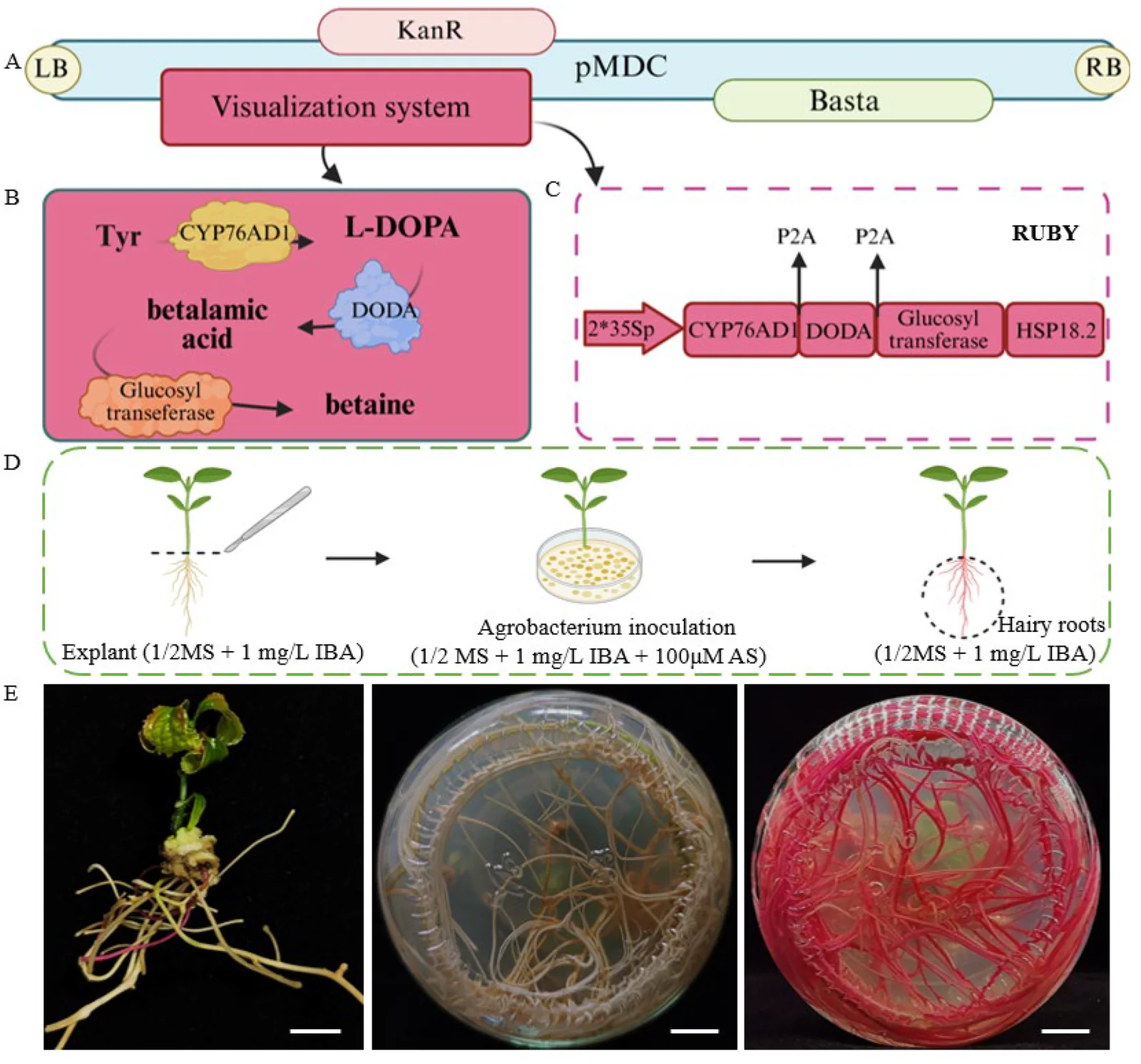
Principle, structure and application of RUBY. (**A**) Schematic representation of the integrated visualization system. Based on the plant binary expression vector pMDC backbone, harboring kanamycin resistance (KanR) and Basta resistance selection markers. LB, left border; RB, right border. (**B**) The visualization system (RUBY) synthesizes the natural product betalain from tyrosine as a substrate by the catalytic action of three enzymes, CYP76AD1, DODA and Glucosyl transferase. (**C**) Schematic diagram of the RUBY element structure, driven by the 2 × 35S promoter. The *CYP76AD1*, *DODA* and glycosyl transferase genes are linked via the P2A peptide, and HSP18.2 is the transcription terminator. (**D**) Schematic diagram of the *Agrobacterium*-mediated hairy root transformation process in *E. ulmoides*. (**E**) Phenotypic comparison of RUBY-expressing hairy roots. Left: composite plant with chimeric hairy-roots; middle: wild-type hairy roots; right: RUBY-positive hairy roots (RUBY+). Arrows indicate the direction of biochemical reactions and experimental workflows. Colored boxes distinguish different functional modules. Bar = 1 cm.

To address the cumbersome screening process in *E. ulmoides* hairy root transformation, we first constructed a visual selection vector based on the RUBY reporter system. The pMDC vector was selected as the backbone, which contains T-DNA borders (LB/RB), a bacterial selection marker (Kan) and a plant selection marker (Basta) (Figure 1A). The visualization system was designed to convert tyrosine in plants into betalains through a three-step enzymatic reaction involving *CYP76AD1*, *DODA*, and a *glucosyltransferase* (Figure 1B). In the RUBY expression cassette, the three genes were fused via P2A peptides and driven by a double *CaMV 35S* promoter with the Hsp18.2 transcription terminator, enabling the coordinated expression of three enzymes (Figure 1C). To verify whether this construct could be stably expressed in *E. ulmoides* hairy roots, we used rootless *Eucommia* seedlings as explants and performed transformation via *Agrobacterium*-mediated infection (Figure 1D). The results showed that transgenic hairy roots harboring the RUBY expression cassette exhibited a distinct red color, while non-transformed roots remained white or light-colored. Moreover, the red phenotype of the transgenic hairy roots remained stable during growth and did not fade or disappear over time. Under the same cultivation conditions, there was no visible difference in the growth status between the RUBY-positive hairy roots and the wild-type hairy roots (Figure 1E). Furthermore, we compared total triterpenoid content between wild-type and RUBY-positive hairy roots, and no significant difference was observed (Figure S1). These results indicated that the RUBY system enables rapid and reliable visual screening of transgenic hairy roots without additional staining or fluorescence detection, and can be stably expressed during plant growth without affecting their triterpenoid accumulation.

### 2.2. Optimization of Transformation Conditions for Hairy Roots in E. ulmoides

**Figure 2:**
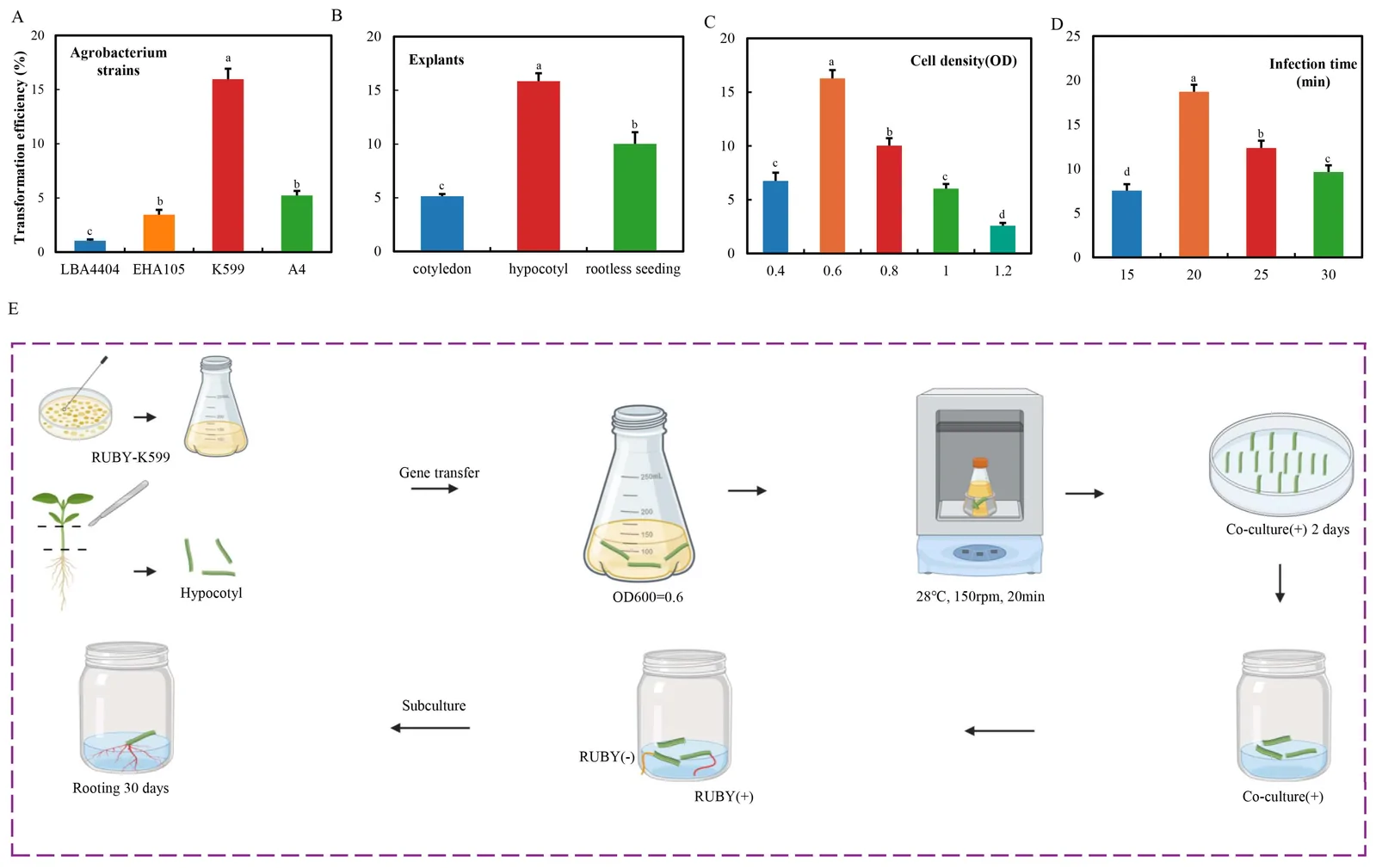
Optimization and development of a hairy root genetic transformation system in *E. ulmoides*. (**A**) Influence of different *Agrobacterium* strains (LBA4404, EHA105, K599 and A4) on transformation efficiency. (**B**) Influence of different explant types (cotyledon, hypocotyl, and rootless seedling) on transformation efficiency. (**C**) Influence of different *Agrobacterium* concentrations (OD_600_ = 0.4, 0.6, 0.8, 1.0 and 1.2) on transformation efficiency. (**D**) Influence of different infection times (15, 20, 25 and 30 min) on transformation efficiency. Transformation efficiency (%) = (Number of RUBY-positive hairy roots/Total number of hairy roots) × 100. Different letters indicate significant difference (*p* < 0.05). (**E**) The schematic diagram of the optimized *Agrobacterium*-mediated hairy root transformation process in *E. ulmoides*. Hypocotyl explants were inoculated with *Agrobacterium* K599 strain (OD_600_ = 0.6) harboring the RUBY vector for 20 min, and subsequently co-cultured for 2 d. After co-cultivation, the hypocotyl explants were move to selection medium. The RUBY-positive explant was selected and subjected to expanded culture.

To enhance the induction efficiency of transgenic hairy roots, we compared and analyzed the effects of *Agrobacterium* strain, explant type, infection concentration, and infection time on transformation efficiency. Using the previously validated visual gene RUBY as a selection marker, we first compared the effects of four *Agrobacterium* strains (including LBA4404, EHA105, K599 and A4) on the transformation efficiency of transgenic roots in *E. ulmoides* (Figure S2A). The results showed that the strain K599 achieved the highest transformation efficiency of 15.96 ± 0.64%, which was significantly higher than other strains. Among these, the strain LBA4404 showed the lowest transformation efficiency of 1.05 ± 0.33% (Figure 2A; Table 1). Therefore, the K599 strain was selected as the optimal strain for further optimization of other parameters affecting transformation efficiency. Subsequently, the transformation efficiency of different *E. ulmoides* explant types was compared. All explants (cotyledon, hypocotyl and rootless seedling) produced hairy roots after *Agrobacterium* K599-mediated transformation (Figure S2B). Among the selected *E. ulmoides* explants, the hypocotyl segments exhibited the highest transformation efficiency (15.85 ± 0.53%). Although rootless seedlings showed a higher hairy root induction rate (64.30 ± 6.50%), the transgenic hairy root rate was lower than that of hypocotyls (10.02 ± 0.89%). The cotyledons showed the lowest transformation efficiency among the three explants (5.15 ± 0.50%; Figure 2B and Figure S2C). Thus, we used the *E. ulmoides* hypocotyls as materials to determine the optimal concentration and infection time for *Agrobacterium* infection.

To further evaluate the influence of *Agrobacterium* concentration on the transformation efficiency of transgenic roots in *E. ulmoides*, we established five different concentrations (OD_600_ = 0.4, 0.6, 0.8, 1.0 and 1.2) and compared the transformation efficiencies across these treatments. The highest transformation efficiency was observed at an OD_600_ of 0.6. At this concentration, the induction rate (44.25 ± 2.84%) of *E. ulmoides* hairy roots was the highest, and the number of RUBY-positive hairy roots was also the largest (Figure 2C and Figure S3A). Moreover, when the bacterial concentration was further increased, the transformation efficiency decreased significantly (Figure S3B). Based on these results, we determined that an OD_600_ of 0.6 was the optimal infection concentration and was used for subsequent research. Finally, we established four different time treatments (15, 20, 25, and 30 min) and compared the transformation efficiencies to assess the impact of *Agrobacterium* infection time. The results showed that the transformation efficiency first increased and then decreased with prolonged infection time (Figure 2D). The highest transformation efficiency was achieved at an infection time of 20 min (18.71 ± 0.81%), which was significantly higher than that of other groups. When the infection time was increased to 25 and 30 min, the transformation efficiency decreased significantly (12.36 ± 0.65% and 9.65 ± 0.88%, respectively). The infection time of 15 min exhibited the lowest transformation efficiency (7.54 ± 0.93%). According to the above results, we determined 20 min as the optimal *Agrobacterium* infection time (Table 2). Collectively, these results systematically optimized the key parameters of the *Agrobacterium*-mediated transformation system for *E. ulmoides* hairy roots. *Agrobacterium* strain K599, hypocotyl explants, an OD_600_ value of 0.6, and a 20 min infection time were determined as the optimal conditions for *E. ulmoides* hairy root transformation.

### 2.3. Establishment of a Hairy Root Genetic Transformation System in E. ulmoides

The major difficulty in genetic transformation of *E. ulmoides* is the low transformation efficiency, which limits its application in genome editing and genetic breeding. Based on the optimization of key parameters, including *Agrobacterium* strain, explant type, bacterial concentration, and infection times (Figure 2A–D), we established an efficient *Agrobacterium*-mediated hairy root genetic transformation system for *E. ulmoides*. In our system, the RUBY reporter gene was used as a selectable marker. Firstly, the hypocotyls of *E. ulmoides* seedlings grown for about 40 d were selected as explants for infection. The RUBY expression vector was transformed into *Agrobacterium* strain K599, and the bacteria were cultured to an OD_600_ of 0.6. Next, the hypocotyl explants were incubated with the bacterial suspension and shaken for 20 min at 28 °C and 150 rpm to allow adequate bacterial adhesion and infection. To promote T-DNA integration, the explants were first co-cultivated in darkness for 2 d. After co-cultivation, they were transferred to a rooting medium and cultured for 30 d. During the period of rooting culture, the RUBY reporter system allowed visual selection of positive transformants. The explants which produced bright red hairy roots were identified as RUBY-positive transgenics, while those with white roots were considered non-transgenic (Figure S4). Finally, the RUBY-positive hairy roots were selected and sub-cultured to obtain abundant transgenic lines, which provided materials for subsequent functional gene research (Figure 2E). Overall, these results indicated that our optimized transformation system enabled efficient generation of transgenic hairy roots in *E. ulmoides* using the RUBY visual reporter.

### 2.4. Construction of a RUBY-Based CRISPR-PTG Editing Vector Targeting EuSQS in E.ulmoides

**Figure 3:**
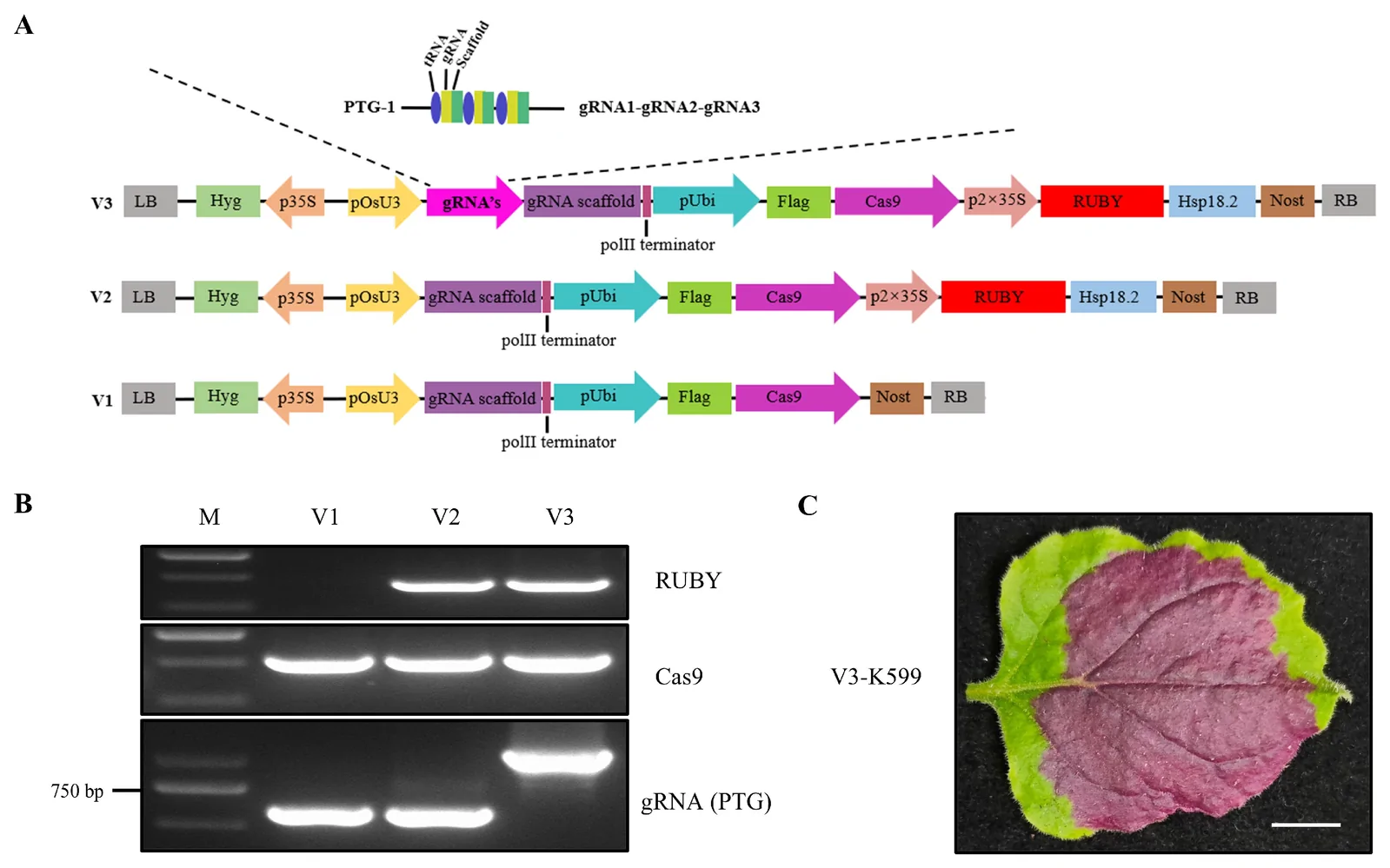
RUBY-based CRISPR-PTG gene-editing system in *E. ulmoides*. (**A**) The structural diagrams of the three vectors used in this study. Vector 1 (V1) contains an expression cassette of Cas9 driven by the UBI promoter, and a genome editing gRNA cassettes (including a pOsU3-gRNA-scaffold-terminator). Vector 2 (V2) incorporates the RUBY reporter gene driven by the 2 × 35S promoter and the Hsp18.2 transcription terminator on the basis of V1. Vector 3 (V3) incorporates the PTG (polycistronic tRNA-gRNA) expression module on the basis of V2. The dashed line indicates the insertion site of the PTG (polycistronic tRNA-gRNA) module into the V3 vector. Hyg, Hygromycin resistance; Flag tag; Nost, nopaline synthase terminator; LB, left border; RB, right border. (**B**) Confirmation of vector construction. The Cas9 gene, RUBY reporter gene and PTG module were detected. M, DNA marker. (**C**) Transient expression assay of *Agrobacterium* K599 harboring V3 in *Nicotiana benthamiana*. Bar = 1 cm.

To assess the feasibility of gene-editing with the *E. ulmoides* genetic transformation system that we established, we designed a CRISPR-Cas9-based gene-editing system containing a polycistronic tRNA-gRNA (PTG) tandem module, which enabled the expression of three gRNAs (gRNA1-gRNA2-gRNA3) targeting the SQUALENE SYNTHASE (*EuSQS*) gene, thereby improving gene editing efficiency. In this part, we constructed three binary expression vectors (V1, V2 and V3) to verify whether the gene-editing system was applicable to the established genetic transformation system of *E. ulmoides* (Figure 3A). V1 was the plasmid pRGEB32, which contained a Cas9 expression element driven by the Ubi promoter and carried a Flag tag used for protein detection as well as a gene (Hyg) for plant-resistance selection (Figure S7A). Notably, (V1) lacked both the RUBY reporter and the polycistronic tRNA-gRNA (PTG) tandem module. To achieve rapid and non-destructive visual screening of positive hairy roots during tissue culture for gene editing, we modified V1 by inserting the RUBY reporter gene which was driven by the 2 × 35S promoter and terminated by the Hsp18.2 terminator (V2).

The SQUALENE SYNTHASE gene (*EuSQS*) was chosen as the target for testing the CRISPR-PTG system. The genomic structure of *EuSQS* was analyzed. The gene contains a total of 13 exons, and three gRNA target sites were designed based on this structure (Figure S8A). The model showed that gRNA1 targeted the first exon, while gRNA2 and gRNA3 targeted the sixth and seventh exons, respectively. Off-target prediction results showed that gRNA1 had no high-risk off-target sites, whereas gRNA2 and gRNA3 were predicted to have 8 and 1 potential high-risk off-target sites, respectively (Table S4). All three gRNAs were perfectly matched to the *EuSQS* target sequences, with no base mismatches (Table S5). This multi-site targeting strategy could improve gene-editing efficiency. Therefore, we constructed a PTG structure using the pGTR vector (Figure S7B) to assemble gRNA1-gRNA2-gRNA3 in tandem (tRNA-gRNA1-tRNA-gRNA2-tRNA-gRNA3). Subsequently, the PTG cassette was cloned into V2 under the control of the OsU3 promoter (Figure 3A, V3).

To verify the modified vector 3 (V3), we first analyzed the key components, including the RUBY reporter gene, the Cas9 gene, and the PTG cassette, by gel electrophoresis. The results showed that all three genes were successfully detected in V3 (Figure 3B). Notably, after we transiently expressed V3 into tobacco leaves via *Agrobacterium*, the leaves showed a bright red color, indicating that the RUBY reporter gene in V3 could be stably expressed (Figure 3C). Collectively, these results demonstrated that we successfully constructed a RUBY-based CRISPR-PTG gene-editing vector system targeting *EuSQS* in *E. ulmoides*.

### 2.5. Analysis of Transgenic Hairy Root Screening and Transformation Efficiency

**Figure 4:**
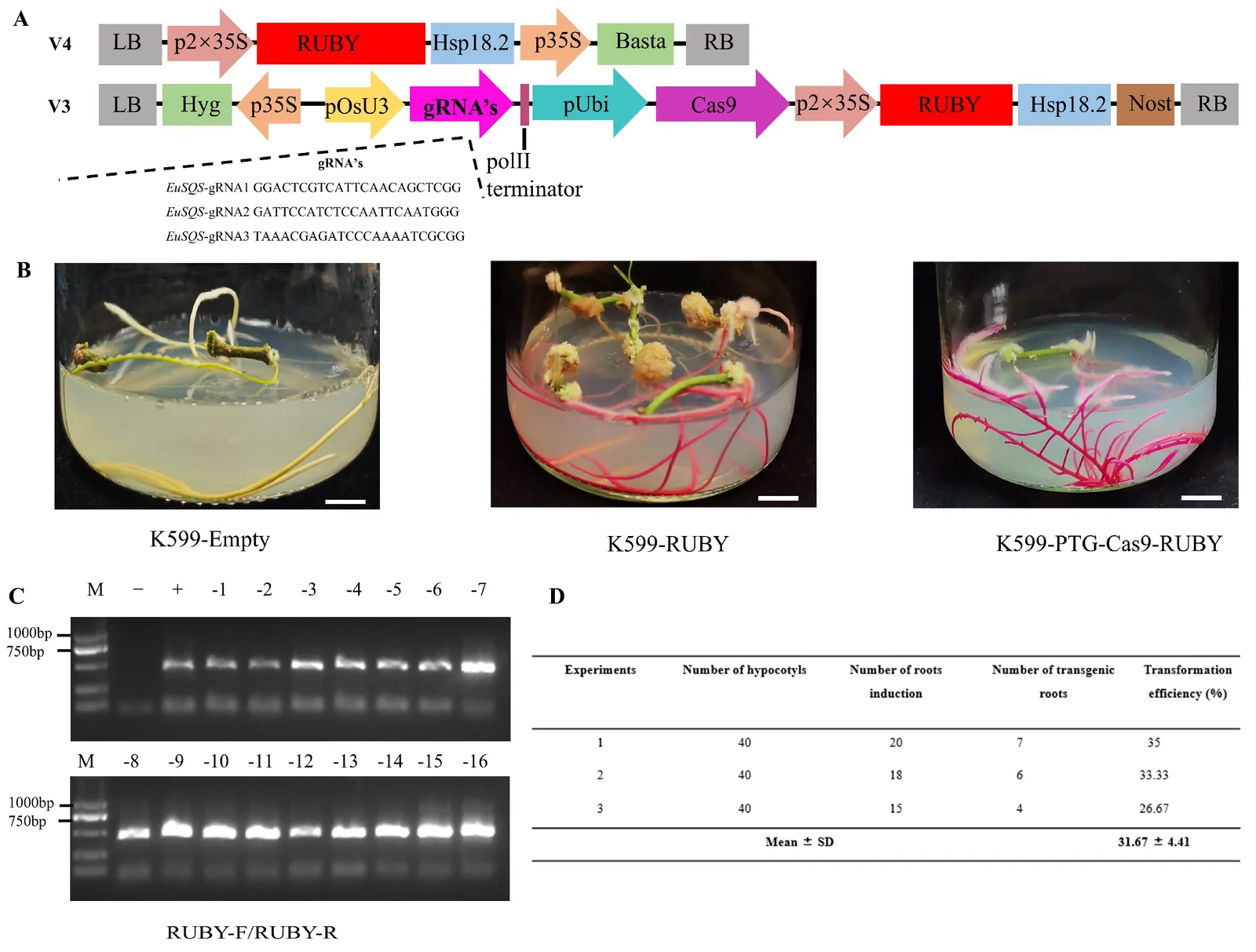
Establishment and verification of the CRISPR-PTG-RUBY gene-editing system in *E. ulmoides* hairy roots. (**A**) Schematic diagram of the vector structure. Vector 4 (V4) is a control vector that only harbors the RUBY reporter gene. Vector 3 (V3) is a CRISPR-PTG-RUBY vector that harbors three gRNAs targeting the *EuSQS* gene. The dashed line indicates the three linked gRNA sequences. (**B**) Phenotypic analysis of hairy roots. K599-Empty, no red phenotype; K599-RUBY, red phenotype; K599-PTG-Cas9-RUBY, red phenotype. Bar = 1 cm. (**C**) PCR identification of transgenic hairy roots. M, marker; -, wild-type root; +, V3; 1–16, independent hairy root lines. (**D**) Transgenic ratio of RUBY-positive hairy roots.

To verify the reliability of the RUBY visual screening system and the stable expression of the RUBY-based CRISPR-PTG editing vector in *E. ulmoides* hairy roots, we performed a comparative analysis of the expression vectors. V4 contained only the RUBY reporter gene as a control, and V3 contained both the RUBY reporter gene and the PTG module (gRNA1-gRNA2-gRNA3) targeting *EuSQS* (Figure 4A). Subsequently, the vectors were introduced into *Agrobacterium* K599, and the hypocotyls were transformed using the established transformation protocol for *E. ulmoides*. The phenotypes of the hairy roots were compared. In the control group (empty), only white roots were observed, whereas the positive roots carrying RUBY (V4) or PTG-RUBY (V3) showed a bright red color (Figure 4B). We also detected the RUBY gene by PCR in red root samples carrying V3. All samples yielded the expected size bands, which indicated that the RUBY system was suitable for identifying transgenic positive hairy roots in *E. ulmoides* (Figure 4C). Moreover, we statistically analyzed the proportion of red positive hairy roots. The average transformation efficiency was 31.67 ± 4.41% (Figure 4D). These results demonstrated that the RUBY-based CRISPR-PTG system enabled efficient screening of transgenic *E. ulmoides* hairy roots, offering a new tool for subsequent gene editing.

### 2.6. CRISPR-PTG-Mediated Genome Editing of EuSQS in E. ulmoides Hairy Roots

**Figure 5:**
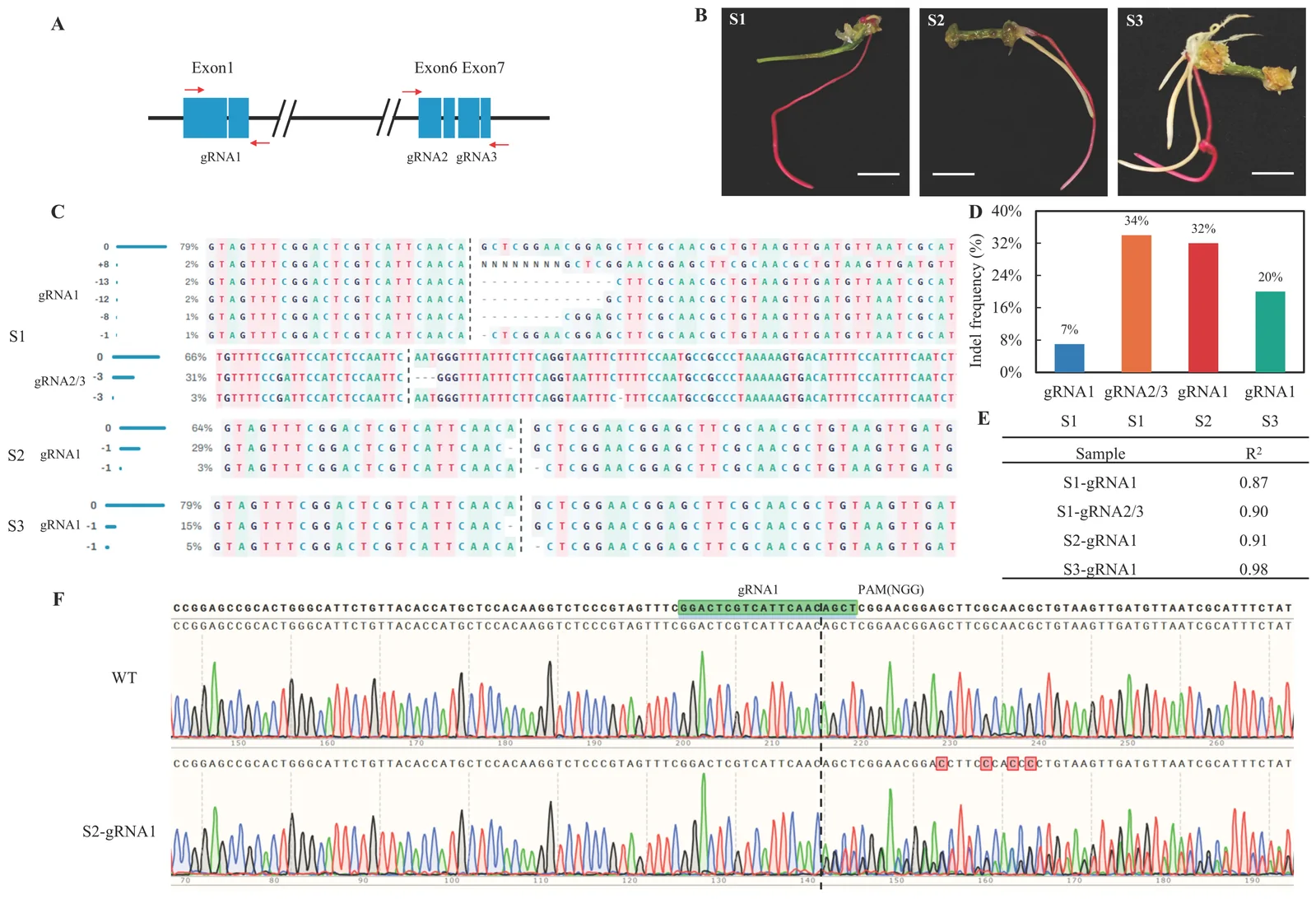
CRISPR-PTG-Cas9 mediated gene editing in *E. ulmoides* hairy roots. (**A**) Positions of gRNA target sites in the *EuSQS* gene and gene-specific primers used for sequencing are indicated by arrows (red). (**B**) Phenotypes of positive hairy root samples (S1, S2, S3). Bar = 1 cm. (**C**) Indel profiles at the target sites in three samples analyzed by ICE (Inference of CRISPR Editing, version 4.2) software. The gRNA1 target region was analyzed in all three lines, whereas the gRNA2-gRNA3 region was analyzed only in S1. The length of blue bars indicates the relative abundance (frequency) of each indel variant. (**D**) Indel frequencies at different target sites in different hairy root samples. (**E**) The coefficient of determination (R^2^) values from ICE analysis indicate that the Sanger sequencing data used for Indel inference were of high quality. (**F**) Sanger sequencing chromatograms of the gRNA1 target site in Sample 2 (S2) compared with the wild type (WT). The green box indicates the gRNA1 sequence, and the PAM (NGG) motif is labelled above the sequence.

To assess the feasibility of CRISPR-PTG-mediated genome editing in *E. ulmoides* hairy roots, we constructed a multiplex CRISPR-Cas9 system targeting the *EuSQS* gene. Three independent PCR-confirmed RUBY-positive red hairy root lines (lines 9, 10, and 15, renamed as S1, S2 and S3, respectively) carrying the V3 were selected for subsequent editing analysis (Figure 5B). Due to the short distance between gRNA2 and gRNA3 (158 bp), we sequenced the gRNA1 and gRNA2-gRNA3 regions of the *EuSQS* gene to analyze gene-editing events (Figure 5A). The PCR products that contained gRNA target regions from the amplified samples were individually sequenced. The gRNA1 target region was analyzed in all three lines, whereas the gRNA2-gRNA3 region was analyzed only in S1 (Figure 5D). ICE analysis showed that in sample S1, the indel frequency at the gRNA1 site was 7% (R^2^ = 0.87), while that at the gRNA2-gRNA3 dual-target region was 34% (R^2^ = 0.90). The indel frequencies at the gRNA1 site in sample S2 and sample S3 were 32% (R^2^ = 0.91) and 20% (R^2^ = 0.98), respectively. The R^2^ value measures the consistency between the indel distribution predicted by ICE and the Sanger sequencing data; higher values indicate greater confidence in the estimates. All R^2^ values exceeded 0.87, indicating a good fit between the ICE model and the Sanger sequencing traces, thereby supporting reliable estimation of indel frequencies (Figure 5C–E). ICE analysis predicted that the detected editing events were predominantly small deletions, with −1 bp deletions being the most frequent. The results showed that the indel frequencies differed among different target regions and independent hairy root lines, which indicated that CRISPR-Cas9-mediated gene-editing events had occurred.

To further examine the editing authenticity, we analyzed the Sanger sequencing results of the *EuSQS* target region. Compared with the wild type, the gRNA1 of S2 showed obvious overlapping peaks at the target site region, and these overlapping peaks started from the Cas9 cleavage site, indicating that the CRISPR-PTG system successfully induced genome editing at the gRNA1 target site (Figure 5F). In S1, overlapping peaks were observed within the gRNA2-gRNA3 target region by Sanger sequencing, suggesting the presence of CRISPR-induced mutations (Figure S5A). S3 and S2 exhibited similar Sanger sequencing chromatograms at the gRNA1 target site, but differed in both editing efficiencies and editing outcomes (Figure 5F and Figure S5B). The raw Sanger chromatograms for all target regions were provided in the Supplementary Material. Additionally, two high-risk off-target sites of gRNA2 and one high-risk off-target site of gRNA3 with higher predicted risk scores were selected and analyzed through Sanger sequencing and ICE detection in transgenic hairy roots (Table S7). The results showed that the indel mutation frequency at both tested sites was 0% in all samples, indicating that no off-target mutations were detected. Meanwhile, we compared the betacyanin accumulation levels between RUBY-positive hairy roots (without PTG) and line S2 (with PTG), and no significant difference was observed (Figure S6). Taken together, these results provide preliminary evidence that CRISPR-PTG-mediated targeted editing can occur at the *EuSQS* locus in *E. ulmoides* hairy roots.

## 3. Discussion

In this study, we successfully established an efficient, stable and visual screening system for *E. ulmoides* hairy roots based on the RUBY reporter system combined with an optimized *Agrobacterium*-mediated transformation protocol (Figure 2E). Furthermore, we constructed a RUBY-based CRISPR-PTG multiplex gene-editing system and verified its feasibility for gene editing in *E. ulmoides* hairy roots by using the *EuSQS* gene as the target (Figure 3, Figure 4 and Figure 5). In summary, this study provides a reliable platform for gene function research and molecular breeding of *E. ulmoides*, thereby offering important support for improving its economic and medicinal value.

### 3.1. The RUBY Reporter System Enables Non-Destructive and Visual Screening of Transgenic Hairy Roots

In this study, the RUBY reporter system based on the betalain synthesis pathway was applied to transforming *E. ulmoides* hairy roots. Compared with traditional fluorescent proteins or GUS reporter systems, RUBY enables rapid and non-invasive screening of transgenic tissues by accumulating visible red betalains, without the need for exogenous substrates or expensive equipment [27]. The results showed that the RUBY reporter gene exhibited a stable bright red color in positive transgenic hairy roots of *E. ulmoides*, which was consistent with previous research in other woody plants [8,28,29]. Moreover, the betalain biosynthesis pathway from tyrosine is metabolically compatible in the plant cell environment and has no significant impact on the growth of plant roots [13]. Consistently, RUBY-positive hairy roots showed no obvious visual growth difference under the same cultivation conditions. The comparison of total triterpenoid content in hairy roots showed that RUBY introduction did not significantly interfere with secondary metabolism in *E. ulmoides* hairy roots. This confirms that RUBY can serve as a universal and convenient selection marker for woody plants difficult to transform [30,31]. Therefore, this study established RUBY as a visual marker for root transformation in *E. ulmoides* and successfully applied it to *E. ulmoides* hairy roots. However, this study did not conduct quantitative evaluations of fresh weight, growth rate, and branching characteristics. The potential impact of these factors on the development and metabolism of hairy roots still requires further investigation. Taken together, the application of the RUBY reporter system in this study optimizes the screening procedure for transgenic hairy roots of *E. ulmoides* and provides an efficient screening tool for functional gene research in this medicinal plant.

### 3.2. Optimization of Key Parameters for Hairy Root Transformation

As shown in Table S3, the parameters of RUBY-mediated transformation vary greatly among woody plants [13,30,31,32,33]. *E. ulmoides* is an important medicinal woody plant rich in various secondary metabolites [34]. At present, tissue culture of *E. ulmoides* is one of the important technical approaches for related research [35,36,37]. The transformation method mediated by *Agrobacterium* is the main strategy for exploring gene function in *E. ulmoides* [35,38]. However, the current regeneration system is still limited to the induction and cultivation of callus tissue [39,40], which also restricts functional studies of *E. ulmoides*. Based on the *Agrobacterium*-mediated genetic transformation method [41], we systematically optimized and revealed the key factors in the transformation process of *E. ulmoides*. Among the four selected *Agrobacterium* strains [42], the K599 strain exhibited the highest transgenic root induction efficiency, which was consistent with the research results in plants such as poplar and cotton [43,44]. This was closely associated with the strong hairy root induction ability of strain K599, which harbored the Ri plasmid [45]. The explant type also significantly influenced the transformation efficiency [46]. We found that the hypocotyl exhibited a significantly higher transformation efficiency. The tissue culture of *E. ulmoides* also mostly used the hypocotyl as the explant [47]. This was because the cells in the hypocotyl of *E. ulmoides* had stronger cell-division and tissue-regeneration capabilities, and were easier to accept and integrate the exogenous T-DNA. Studies have shown that low bacterial concentrations reduce the contact between *Agrobacterium* and the explant, while an excessive concentration easily induces browning, thereby affecting the efficiency of hairy root induction [48]. After optimization, the optimal infection concentration was OD_600_ = 0.6. Moreover, infection time is one of the key factors affecting transformation efficiency. A short time is unfavorable for T-DNA integration while an excessively long incubation time results in overgrowth of *Agrobacterium*, leading to death of explants [49]. We ultimately determined that the optimal time for *Agrobacterium* infection is 20 min.

These results demonstrate that genetic transformation conditions are crucial for transformation efficiency. Therefore, through a series of process optimizations, we established a complete and efficient genetic transformation protocol for generating *E. ulmoides* hairy roots. Although this protocol cannot regenerate complete transgenic *E. ulmoides* plants, it overcomes the previous limitation of callus-only research and provides a standardized and reliable technical system for dissecting gene function in this species.

### 3.3. RUBY-Based CRISPR-PTG Gene-Editing System

The PTG strategy expresses multiple gRNAs through a single transcript, thereby coordinately guiding Cas9 to recognize and cleave multiple target sites, ultimately improving the editing efficiency of a single gene. The production of polycistronic gRNAs (PTG) using the endogenous tRNA system has been demonstrated to significantly enhance target editing efficiency in various plants such as rice and *Arabidopsis* [16,50]. Based on the established genetic transformation system of *E. ulmoides* hairy roots, we further developed a RUBY-based CRISPR-PTG gene-editing system and selected *EuSQS* as the target gene. The results clearly showed that targeted mutations were induced in the *E. ulmoides* roots, and were verified by Sanger sequencing and ICE analysis. Notably, there were differences in the editing efficiency among different gRNA target sites, which were consistent with previous findings. This may be related to the differences in the gRNA sequences themselves and the processing and release efficiency of gRNAs in the PTG system [28,51,52]. Furthermore, hairy roots generated by *Agrobacterium*-mediated transformation are not typically derived from a single cell but consist of chimeric tissues composed of cells with different editing states, meaning that different cells within the same hairy root may carry different mutations. This is likely to be one of the main reasons for the differences in editing efficiency detected through ICE analysis [53,54].

In this study, the editing analysis was mainly based on Sanger sequencing and ICE analysis of the PCR products. Due to the absence of amplicon deep sequencing and clone amplicon sequencing, the indel events predicted by the ICE analysis cannot be accurately resolved and characterized at the single allele level in terms of the mutation spectrum and frequency. Nevertheless, this study conducted off-target prediction and experimental validation of the predicted high-risk sites, and no indels were detected at any of the tested sites, indicating that off-target effects were unlikely to compromise the gene-editing results. Taken together, the sequencing and ICE analysis obtained in this study provide preliminary evidence that CRISPR-PTG-mediated targeted editing can occur at the *EuSQS* locus in *E. ulmoides* hairy roots. Although these edits occurred in a chimeric hairy root background, the detection of the targeted mutations supported the feasibility of CRISPR-PTG-mediated genome editing in *E. ulmoides*. Notably, RUBY was used only as a qualitative visual marker to identify transgenic hairy roots based on red pigmentation in this study, and betacyanin accumulation levels in the edited hairy roots showed no significant changes. We did not investigate the potential correlation between red color intensity and genome-editing efficiency or chimerism rate. The intensity of red color is not a quantitative indicator of editing activity, given the chimeric nature of hairy roots and the variability in transgene expression and tissue composition. Therefore, future work is needed to determine whether the intensity of RUBY pigmentation correlates with the genome-editing efficiency.

## 4. Materials and Methods

### 4.1. Plant Materials and Growth Conditions

Healthy aseptic seedlings of *E. ulmoides* grown for about 40 d were used as the source of explants in this study. *E. ulmoides* seeds were first vernalized at 4 °C for 2 d, disinfected on the surface with 70% ethanol for 1 min. The seeds were soaked in 5% NaClO solution for 5 min, and then washed five times with sterile water. Under aseptic conditions, the seed ends were excised with aseptic tools and inoculated on solid MS medium. The materials were grown in a light growth chamber at 23 °C, with an 18 h light/6 h dark photoperiod and 60% relative humidity. Different explants (cotyledons, hypocotyls and rootless seedlings) of *E. ulmoides* were selected for subsequent root transformation.

### 4.2. Construction of the RUBY-Based Hairy Root Transformation Vector

The synthesized sequences encoding three betalain biosynthesis genes (*CYP76AD1*, *DODA* and glucosyl transferase) were linked by 2A peptides to form the RUBY expression cassette, which was followed by the transcription terminator Hsp18.2. The fragment was amplified using the primers (Table S2) and then inserted into a binary expression vector based on the pMDC backbone and driven by the *CaMV 35S* promoter via XbaI and EcoRI restriction sites (V4; Figure S8).

For the construction of the plasmid vector pRGEB32-RUBY (V2), the previous plasmid vector V4 was used as a template and the RUBY expression cassette (containing the *CaMV 35S*, RUBY and the Hsp18.2 terminator) was amplified. The backbone vector *pRGEB32* was digested with XbaI and PmeI. After double digestion, the amplified sequence was cloned into pRGEB32.

The polycistronic tRNA-gRNA (*PTG*) genes were synthesized by Golden Gate assembly. The *EuSQS* gene sequence was obtained from the *E. ulmoides* genome database (Home—Genome Warehouse). Three target sites located in different exons were selected using a CRISPR design tool (CRISPR-P v2.0). Cas-OFFinder was used to perform on-target and off-target prediction for the three *EuSQS*-targeting gRNAs. The sequences of gRNAs were listed (Table S1). The pGTR plasmid vector was used as the template (Figure S7B), and a polycistronic tRNA-gRNA (PTG) array was constructed following the method described previously [16], with detailed reactions provided in the Supplementary Materials (construction of PTG component by Golden Gate assembly). Subsequently, the plasmid vector pRGEB32-RUBY containing the Cas9 expression cassette driven by the UBI promoter was digested with BsaI, and the PTG expression cassette obtained after FokI digestion was cloned into the linearized vector and driven by the OsU3 promoter. The resulting plasmid vector was named pRGEB32-PTG-RUBY (V3).

### 4.3. Agrobacterium Transformation and Culture

Four *Agrobacterium* strains (LBA4404, EHA105, K599 and A4) were selected as research subjects, and the RUBY reporter gene was adopted for visual identification of transgenics. The recombinant vector harboring the RUBY reporter gene was introduced into *Agrobacterium* and the cells were cultured at 28 °C for 2 d. A positive single colony was selected and cultured in LB liquid medium containing 50 mg/L rifampicin and 50 mg/L kanamycin. Antibiotics were added after the LB medium had cooled to 50–60 °C. The culture was incubated at 28 °C for 14 h. Next, 2 mL of the bacterial culture was transferred to 100 mL of fresh LB medium and incubated until the OD_600_ reached 1.2.

### 4.4. Preparation of Explants and Agrobacterium-Mediated Transformation

The cotyledons, hypocotyls (approximately 1 cm in length), and roots of aseptic *E. ulmoides* seedlings were excised in a laminar flow hood. A few shallow cuts were made on the surface of the explants using a blade to increase the contact area between the bacterial suspension and the wound sites. The entire transformation process consisted of five consecutive stages, including pre-culture, infection, co-cultivation, root induction and selection, and subculture. For pre-culture, the explants were evenly placed on pre-culture solid medium (1/2 MS supplemented with 30 g/L sucrose, 7.0 g/L agar, and 1.0 mg/L indole-3-butyric acid (IBA), pH 5.8) and cultured at 23 °C in the dark for 2 d. For the *Agrobacterium* infection stage, *Agrobacterium* strains harboring the binary vectors were cultured in LB liquid medium with antibiotics at 28 °C and 200 rpm until the OD_600_ reached 1.2. The bacterial cells were collected by centrifugation at 5000 rpm for 8 min at 28 °C and resuspended in infection buffer (pH 5.8) containing 10 mM MES, 10 mM MgCl_2_, and 200 μM acetosyringone to an OD_600_ of 0.6. Subsequently, the pre-cultured explants were transferred into the adjusted *Agrobacterium* suspension and incubated at 28 °C with agitation at 150 rpm for 20 min. After infection, the residual bacterial suspension on the surface of the explants was removed with sterile filter paper. Then, the explants were inoculated onto co-culture medium (1/2 MS supplemented with 30 g/L sucrose, 7.0 g/L agar, 1.0 mg/L IBA, and 100 µM acetosyringone, pH 5.8) and cultured at 28 °C in the dark for 2 d. After co-cultivation, the explants were transferred to root induction and selection medium (1/2 MS supplemented with 30 g/L sucrose, 7.0 g/L agar, 1.0 mg/L IBA, and 200 mg/L cefotaxime) and cultured at 23 °C under an 18 h light/6 h dark photoperiod for several days until red RUBY-positive hairy roots were generated. The explants containing RUBY-positive hairy roots were selected and transferred to fresh 1/2 MS medium containing 200 mg/L cefotaxime for further root elongation, and sub-cultured every 10 d.

### 4.5. Parameter Optimization for Agrobacterium-Mediated Transformation

After comparing the hairy root induction efficiency of different *Agrobacterium* strains, we selected the optimal strain K599 as the optimal strain and used for subsequent parameter optimization. The main parameters optimized included explant materials of *E. ulmoides*, the bacterial suspension concentration, and the infection treatment time. The cotyledons, hypocotyls, and rootless seedlings of *E. ulmoides* were infected under the same treatment conditions. For each explant type, 40 samples were used with three biological replicates. After 30 d, the transformation efficiency of hairy roots was calculated to determine the optimal explant type. For the bacterial suspension concentration, the previously identified optimal explant material was used. Five concentration gradients (OD_600_ = 0.4, 0.6, 0.8, 1.0 and 1.2) of the *Agrobacterium* suspension were set, and the explants were infected under the same conditions. The optimal bacterial concentration for infection was then determined following the same procedure as described above. For the infection treatment time, four different infection durations (15, 20, 25 and 30 min) were tested. The explants were treated with the optimal bacterial suspension concentration under the same conditions, and the transformation efficiencies were statistically compared to determine the optimal infection time. All optimization experiments were performed with 40 explants per treatment and three independent biological replicates. The parameters were calculated as follows: Hairy root induction rate (%) = (Number of explants producing hairy roots/Total number of explants) × 100. Transformation efficiency (%) = (Number of RUBY-positive hairy roots/Total number of hairy roots) × 100. The average length of hairy roots was measured and expressed as mean ± SD (cm).

### 4.6. Phenotypic Analysis and Molecular Identification of Transgenic Hairy Roots

Phenotypic analysis of the induced *E. ulmoides* hairy roots was performed with the RUBY reporter gene, and transgenic roots could be directly observed as bright red. The red roots were selected and their genomic DNA was extracted for molecular identification. The genomic DNA of *E. ulmoides* hairy roots was extracted with a plant DNA extraction kit (Tiangen, Beijing, China) following the manufacturer’s protocol. The RUBY reporter gene was used as a marker, and the primers were designed (Table S2). The expression of RUBY was detected via PCR amplification and agarose gel electrophoresis.

### 4.7. Analysis of Gene-Editing Efficiency

The *EuSQS* genomic sequence was used as a reference, and specific primers were designed near the gRNA target sites (Table S2). The target fragments were amplified, and the PCR products were purified after agarose gel electrophoresis and subjected to Sanger sequencing. The online tool ICE (Inference of CRISPR Editing) was used to quantify the genome editing efficiency of CRISPR-Cas9, and the R^2^ value was applied to assess the reliability of the sequencing data. Meanwhile, the changes in the Sanger sequencing chromatograms near the Cas9 cut site were analyzed to confirm the occurrence of CRISPR-mediated editing events.

### 4.8. Quantification of Betacyanin Content

Hairy root powder (50 mg) was weighed and added to 1 mL of 70% ethanol. The mixture was centrifuged at 1000 rpm for 10 min and the supernatant was collected. Using 70% ethanol as the blank control, absorbance was measured at 538 nm and 600 nm. Each biological sample was subjected to 3 technical replicates. The betacyanin content was calculated according to the formula. Betacyanin (µg/g FW) = ((A_538_ − A_600_) × M × V × 10^6^)/(ε × W). M = 550 g/mol; V, 0.001 L; ε = 6000 M^−1^cm^−1^; W, 0.05 g; FW, fresh weight.

### 4.9. Quantification of Total Triterpenoid Content

The vanillin-perchloric acid colorimetric method was adopted to measure total triterpenoid levels. *E. ulmoides* powder (50 mg) was weighed and placed in 3 mL of ethanol, and subjected to ultrasonic extraction at 50 kHz and 60 °C for 60 min. Then, the supernatant was collected after centrifugation at 8000 rpm for 8 min. A total of 0.3 mL of the supernatant was transferred to an 8 mL centrifuge tube and evaporated to dryness in a 75 °C water bath. Subsequently, 0.25 mL of the newly prepared 0.5 g/L vanillin-acetic acid solution and 0.75 mL of perchloric acid were added to the tube and mixed thoroughly. A UV spectrophotometer was used to record absorbance at 553 nm.

### 4.10. Statistical Analysis of Data

All data are expressed as mean ± SD. Statistical differences between treatments were analyzed by one-way analysis of variance (ANOVA) followed by Tukey’s post-hoc test (*p* < 0.05).

## 5. Conclusions

In conclusion, this study offers two new technical tools for genetic manipulation in *E. ulmoides*. First, we established a visual hairy root genetic transformation system in *E. ulmoides* using the RUBY reporter gene. The optimized system is capable of rapidly and non-destructively identifying transgenic hairy roots based on the visible red pigment. Second, based on this system, a CRISPR-PTG-RUBY construct was developed and tested for targeted mutations in *E. ulmoides* hairy roots, providing proof-of-concept evidence for CRISPR-PTG-mediated editing. However, *Agrobacterium*-transformed hairy roots may exhibit a chimeric nature, meaning that different cells within a single hairy root may have different editing statuses. Therefore, further optimization and validation are still needed to enhance the editing efficiency and applicability of this system. Overall, this study provides a preliminary basis for future functional gene studies of *E. ulmoides*. It also offers a valuable reference for the genetic transformation research of other woody plants.

## Figures and Tables

**Table 1:** Effects of *Agrobacterium* strains on hairy root induction and transformation efficiency in *E. ulmoides* hypocotyl explants.

Strain Type	Hairy Root Induction Rate (%)	Average Length of Hairy Roots (cm)	Transformation Efficiency (%)
EHA105	28.23 ± 0.65	0.45 ± 0.15	3.44 ± 0.98 ^b^
LBA4404	22.06 ± 0.89	0.12 ± 0.09	1.05 ± 0.33 ^c^
A4	30.58 ± 1.58	0.42 ± 0.54	5.23 ± 0.38 ^b^
K599	59.70 ± 2.34	0.97 ± 0.26	15.96 ± 0.64 ^a^

Table 1 Comparison of hairy root transformation parameters among different *Agrobacterium* strains in *E. ulmoides*. Transformation parameters include hairy root induction rate, average length of hairy roots, and transformation efficiency. Data are shown as mean ± SD. Lowercase letters represent significant differences (*p* < 0.05).

**Table 2:** Effects of infection time (min) on hairy root induction and transformation efficiency in *E. ulmoides* hypocotyl explants.

Infection Time (min)	Hairy Root Induction Rate (%)	Average Length of Hairy Roots (cm)	Positive Rate of Hairy Roots (%)
15	45.63 ± 0.07	0.73 ± 0.18	7.54 ± 0.93 ^d^
20	46.89 ± 2.28	0.79 ± 0.05	18.71 ± 0.81 ^a^
25	50.63 ± 1.63	0.82 ± 0.23	12.36 ± 0.65 ^b^
30	47.37 ± 1.34	0.75 ± 0.12	9.65 ± 0.88 ^c^

Table 2 Comparison of hairy root transformation parameters among different infection times in *E. ulmoides*. Transformation parameters include hairy root induction rate, average length of hairy roots, and positive transformation rate. Data are shown as mean ± SD. Lowercase letters represent significant differences (*p* < 0.05).

## Data Availability

The data presented in this study are available within the article and Supplementary Materials.

## Supplementary Materials

The following supporting information can be downloaded at: https://www.mdpi.com/article/10.3390/plants15182795/s1.

## Abbreviations

The following abbreviations are used in this manuscript:

AS	acetosyringone
CRISPR	Clustered regularly interspaced short palindromic repeats
gRNA	guide RNA
ICE	Inference of CRISPR Edits
IBA	indole-3-butyric acid
OD_600_	optical density at 600 nm
PCR	Polymerase chain reaction
PTG	Polycistronic tRNA-gRNA
RUBY	Betalain reporter system
SD	standard deviation
tRNA	transfer RNA
