# Supplementary material for: A RUBY-Based Visual Hairy Root Transformation and CRISPR-PTG Genome-Editing System in Eucommia ulmoides
Source: Plants (Basel). 2026 Sep 11;15(18):2795. doi: 10.3390/plants15182795 (PMC13610796; doi:10.3390/plants15182795)
Supplement: Supplementary file 1 [file plants-15-02795-s001.zip › plants-4505193-supplementary.pdf]

Supplementary Materials

1. Supplementary figures

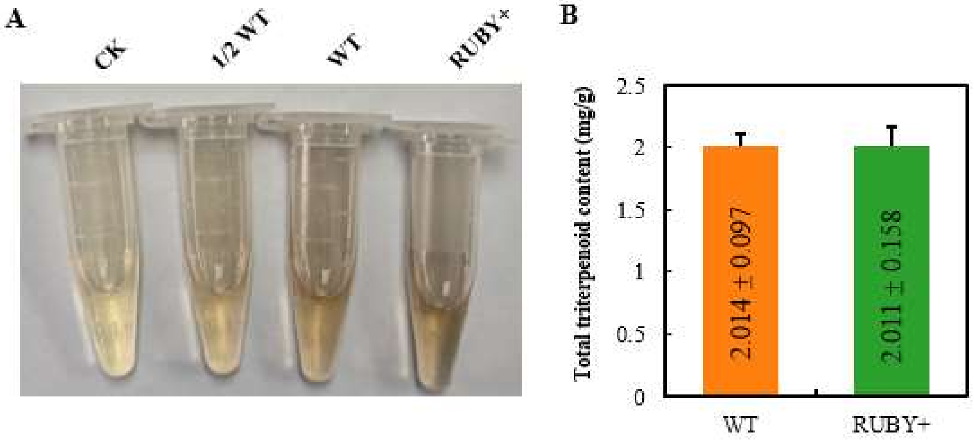

Figure S1. Total triterpenoid content comparison between wild-type and RUBY-positive hairy roots.

(A) Qualitative visual observation of total triterpenoid content. CK, Extraction buffer, control; WT, (wild-type hairy roots); RUBY+ (RUBY-positive hairy roots); (B) Quantitative analysis of total triterpenoid content. WT, (wild-type hairy roots); RUBY+ (RUBY-positive hairy roots). No significant difference between WT and RUBY+.

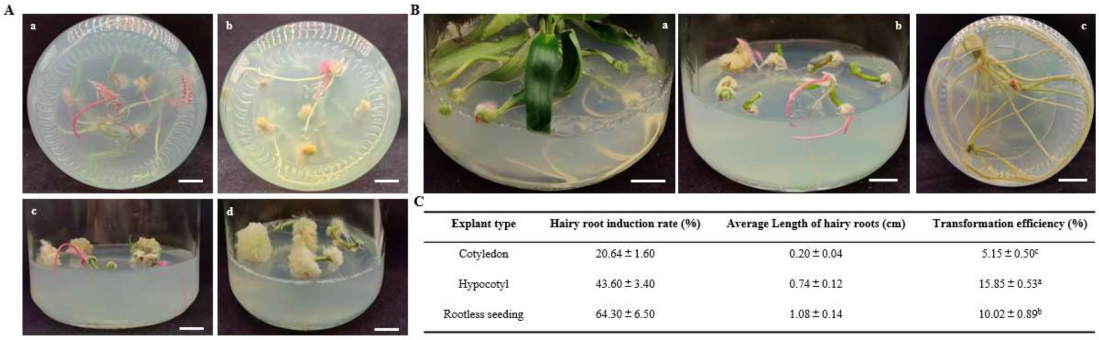

Explant type	Hairy root induction rate (%)	Average Length of hairy roots (cm)	Transformation efficiency (%)
Cotyledon	20.64 ± 1.60	0.20 ± 0.04	5.15 ± 0.50^{c}
Hypocotyl	43.60 ± 3.40	0.74 ± 0.12	15.85 ± 0.53^{a}
Rootless seeding	64.30 ± 6.50	1.08 ± 0.14	10.02 ± 0.89^{b}

Figure S2. Comparison of hairy roots induction from different *Agrobacterium*

strains and *E. ulmoides* explants. (A) (a) K599; (b) A4; (c) EHA105; (d) LBA4404. (B) Phenotypes of hairy roots induced from three types of explants. (a) cotyledon; (b) hypocotyl; (c) rootless seeding. Bar = 1 cm. (C) Statistical analysis of hairy root induction rate, average length of hairy roots, and transformation efficiency. Data are shown as mean ± SD. The lowercase letters represent significant differences ($p < 0.05$).

A

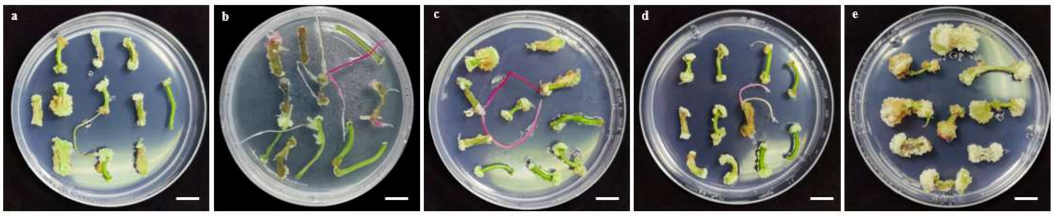

B

Bacterial concentration (OD600)	Hairy root induction rate (%)	Average length of hairy roots (cm)	Transformation efficiency (%)
0.4	50.73 ± 1.36	0.76 ± 0.23	6.75 ± 0.85^{c}
0.6	44.25 ± 2.84	0.77 ± 0.13	16.28 ± 0.46^{a}
0.8	41.63 ± 1.25	0.69 ± 0.14	10.03 ± 0.32^{b}
1.0	36.50 ± 1.83	0.43 ± 0.25	6.03 ± 0.95^{c}
1.2	23.56 ± 0.97	0.26 ± 1.45	2.58 ± 0.41^{d}

Figure S3. Comparison of hairy root induction efficiency under different bacterial concentration in *E. ulmoides*. (A) The phenotypes of hairy roots induced under different *Agrobacterium* concentrations. (a) OD_{600} = 0.4; (b) OD_{600} = 0.6; (c) OD_{600} = 0.8; (d) OD_{600} = 1.0; (e) OD_{600} = 1.2. Bar = 1 cm. (B) Statistical analysis of hairy root induction rate, average length of hairy roots, and transformation efficiency. Data are shown as mean ± SD. The lowercase letters represent significant differences ($p < 0.05$).

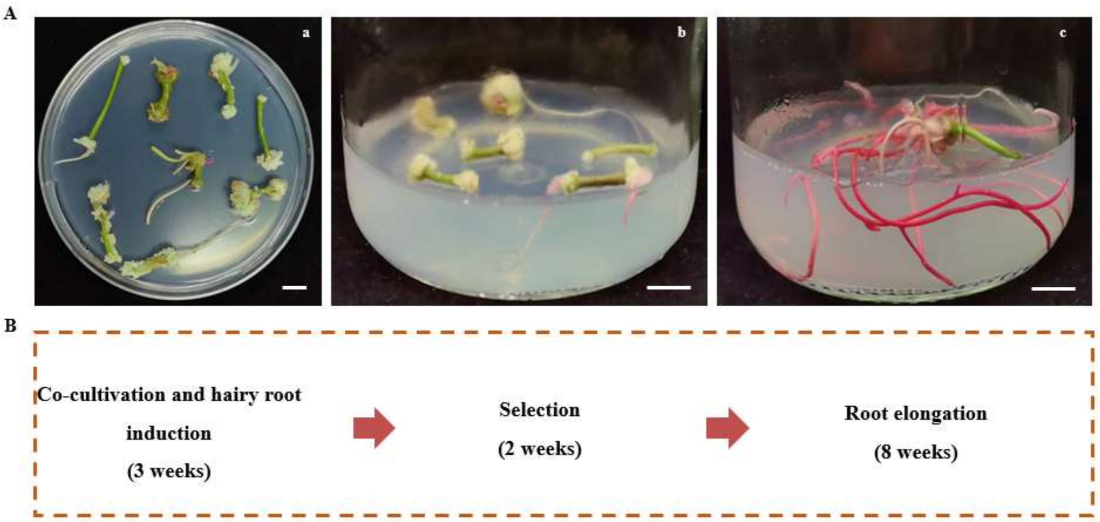

Figure S4. Procedure for hairy-root transformation of *E. ulmoides* using the RUBY reporter gene. (A) The phenotypes at different culture stages of hairy-root transformation. (a) Co-cultivation and hairy-root induction stage (3 weeks); (b) Selection stage (2 weeks); (c) Root elongation stage (8 weeks). (B) Schematic diagram showing the time course of the hairy-root transformation procedure.

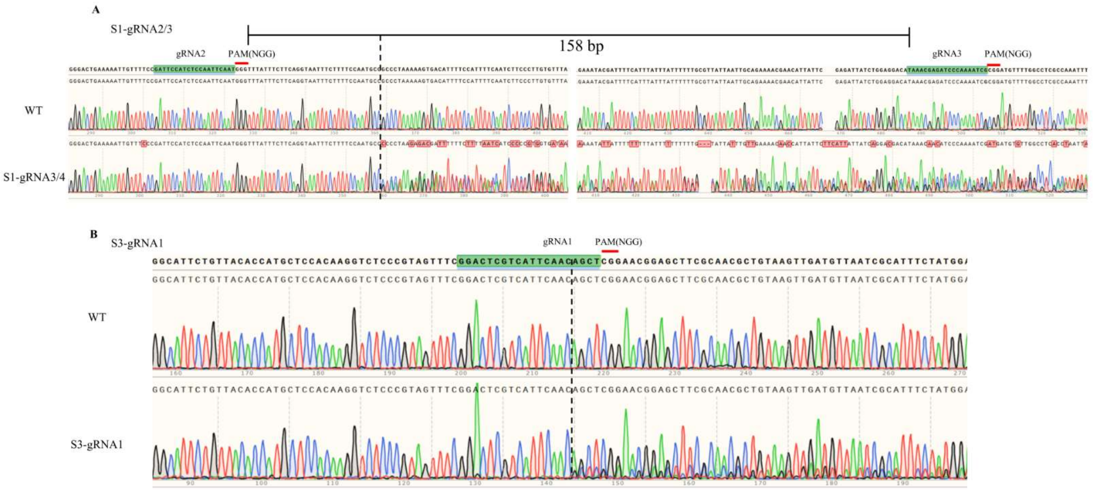

Figure S5. Sanger sequencing chromatograms of the gRNA target site in *E. ulmoides* hairy roots. (A) Sanger sequencing chromatograms of the gRNA2-gRNA3 target site in Sample 1 (S1) compared with the wild type (WT). (B)

Sanger sequencing chromatograms of the gRNA1 target site in Sample 3 (S3) compared with the wild type (WT). Green boxes mark gRNA target sequences, and red bars denote NGG PAM motifs.

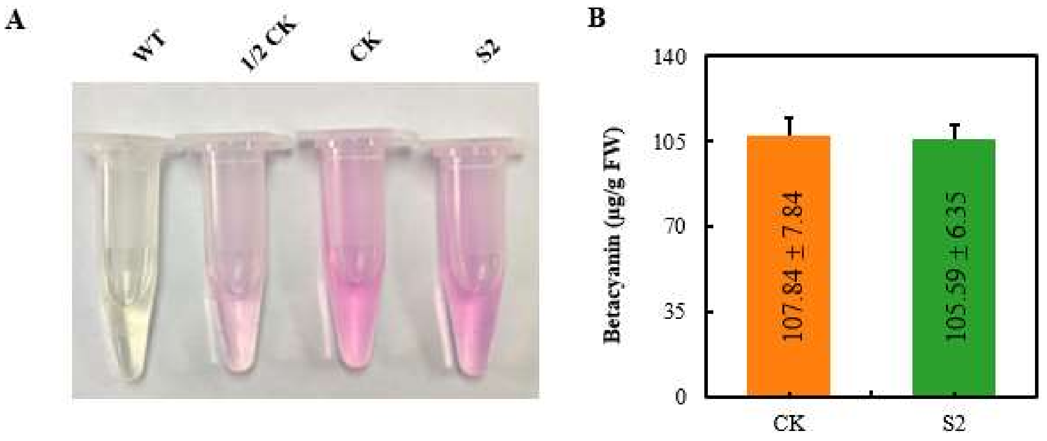

Figure. S6. Comparison of betacyanin content in control and sample 2 (S2). (A) Qualitative visual observation of betacyanin extracts. WT, (wild-type hairy roots); CK, (RUBY-positive control); S2, (sample 2). (B) Quantitative analysis of betacyanin content. CK, (RUBY-positive control, K599-RUBY); S2, (sample 2). No significant difference between WT and RUBY+.

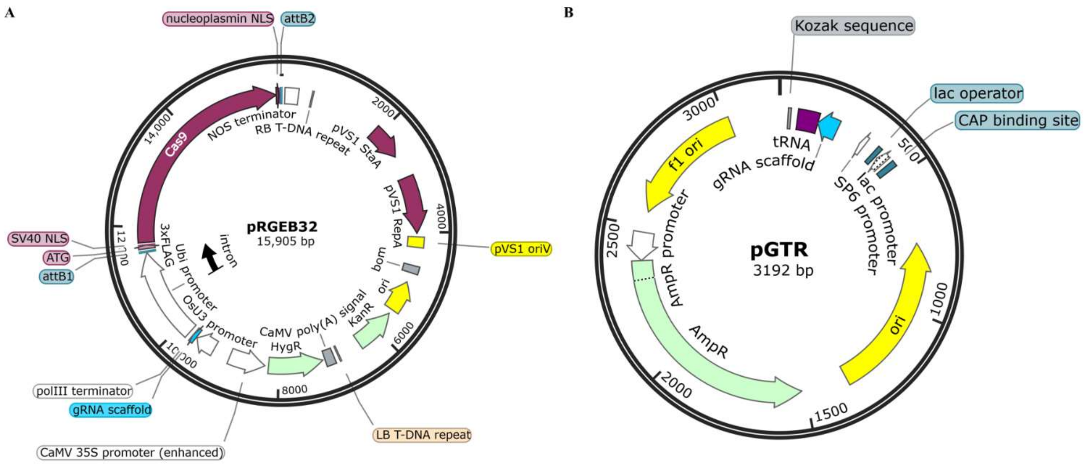

Figure. S7. The maps of the two CRISPR-PTG system vectors. (A) Vector map of pRGEB32 (15 905 bp), which contains *Cas9* expression cassette driven by the Ubi promoter and the *gRNA-scaffold* driven by the OsU3 promoter. Hyg, hygromycin-resistance gene; KanR, Kanamycin-resistance gene; RB, Right border; LB, Left border. (B) Vector map of pGTR (3192 bp), the gRNA-assembly shuttle vector, which contains the *tRNA-gRNA scaffold*. AmpR, ampicillin-resistance gene.

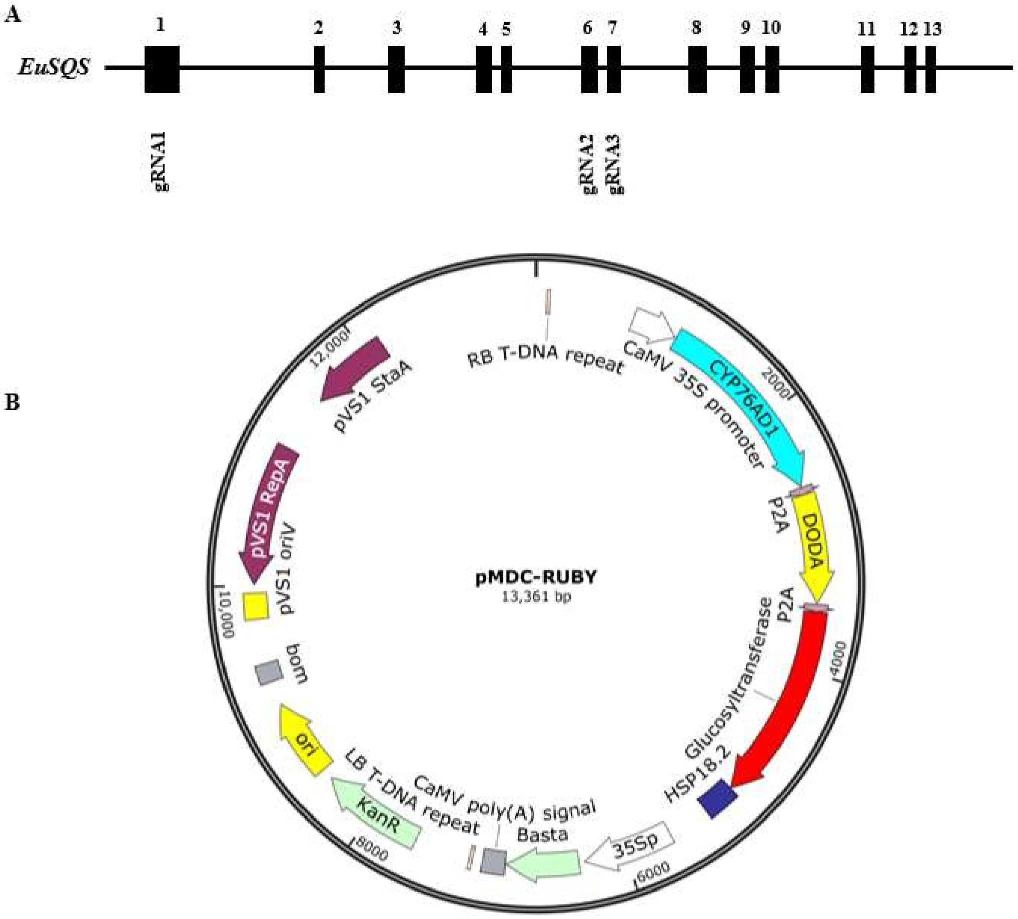

Figure. S8. Gene structure of *EuSQS* and map of the pMDC-RUBY vector (13361 bp). (A) Schematic diagram of the *EuSQS* gene structure and the positions of gRNA target sites. Black boxes represent exons. (B) The RUBY reporter cassette (*CYP76AD1*-P2A-*DODA*-P2A-Glucosyltransferase) is driven by the *CaMV 35S* promoter. Hsp 18.2: the transcription terminator; KanR, Kanamycin-resistance gene; RB, Right border; LB, Left border.

Table S1 The sequence of gRNAs

Name	Sequence (5′-3′)
gRNA1	GGACTCGTCATTCAACAGCTCGG
gRNA2	GATTCCATCTCCAATTCAATGGG
gRNA3	TAAACGAGATCCCAAAATCGCGG

Table S2 The primers used in this study.

Name	Sequence (5′-3′)
gRNA1-F	TA *GGTCTCC* CATTCAACAGCT gttttagagctagaa
gRNA1-R	AT *GGTCTCA* AATGACGAGTCC tgcaccagccgggaa
gRNA2-F	TA *GGTCTCC* CTCCAATTCAAT gttttagagctagaa
gRNA2-R	AT *GGTCTCA* GGAGATGGAATC tgcaccagccgggaa
gRNA3-F	TA *GGTCTCC* ATCCCAAAATCG gttttagagctagaa
gRNA3-R	AT *GGTCTCA* GGATCTCGTTTA tgcaccagccgggaa
L5AD5-F	CG GGTCTC A GGCA GGATG GGCAGTCTG GGCA ACAAAGCACCAGTGG
L3AD5-R	TA GGTCTC C AAAC GGATG AGCGACAGC AAAC AAAAAAAAAA GCACCGACTCG
S5AD5-F	CG GGTCTC A GGCA GGATG GGCAGTCTG GGCA
S3AD5-R	TA GGTCTC C AAAC GGATG AGCGACAGC AAAC
U3P-F	GGGTACGTTGGAAACCACGT
Ubi-R	CCCGGCCCAAATTGAAAAGATAGG
RUBY- XbaI-F	AATTCGCGGCCGCACTCGAGATATCTAGATGAGAC TTTTCAACAAAGGGTAATATCCG
RUBY- PmeI-R	AATATATCCTGTCAAACACTGATAGTTTAAACAAAC AGCTATGACCATGATTACGAAT
RUBY-XbaI-F1	GCTCTAGATGGATCATGCGACCCTCGCCAT

RUBY-EcoRI-R1	CGGAATTCTGGTATGGACTATGGAATATGATTAAAGATAA
RUBY-F1	TAGAAGAAGGCTCGCCTGCT
RUBY-R1	ATCTGGGCATCCTTCGGCACGAT
Cas9-F	CTTCAGCAACGAGATGGCCAAG
Cas9-R	AAGTTGCTCTTGAAGTTGGGGGTC
G1-F	ATGGGGAGTTTGGGGACGCTG
G1-R	AGCTGTTGAATGACGAGTCCGAA
G2-F	ATGACCCCTCTCCACCTAGATTTTC
G3-R	CCAAACAGGGCCTAAGCCTTCTCA

Table S3. Comparison of RUBY-based transformation system in woody plants.

Species	Agrobacterium strain	Explant	Efficiency	CRISPR combined	Reference
Jatropha curcas	K599	cambium	54.18 %	No	Su, Y.; et al. 2026
Plukenetia volubilis	K599	seeding	>18.97	Yes	Lin, K.; et al. 2025
Malus domestica / *Actinidia chinensis*	K599	seeding	regeneration 3.3%	No	Liu, L.; et al. 2024
Citrus	K599	seed	71.4%	Yes	Wang, M.; et al. 2023
Walnut	GV3101	embryo	53.33%	Yes	Song, L.; et al.

					2025
E. ulmoides	K599	hypocotyl	31.67%	Yes	this study

Table S4. High-priority predicted off-target sites of three gRNAs.

This table shows the high-priority off-target candidate sites predicted by Cas-OFFinder (⩽ 3 mismatches, no bulge). Detailed raw data are provided in the accompanying Excel file Table S4_high_priority_off_targets.xlsx.

Table S5. Designed on-target sites of three gRNAs.

This table lists the designed gRNAs and their matching target sites in *E. ulmoides* genome. Detailed raw data are provided in the accompanying Excel file Table S5_on_target.xlsx.

Table S6. Predicted off-target sites of three gRNAs for *E. ulmoides* genome editing.

This table lists the genome-wide off-target prediction results based on Cas-OFFinder in the *E. ulmoides* genome, with CFD scores used to evaluate the Cas9 cleavage risk at each candidate site. Detailed raw data are provided in the accompanying Excel file Table S6_off_target_scored.xlsx.

Table S7. ICE-based experimental detection of predicted off-target sites for gRNA2 and gRNA3.

Sample ID	Predicted off-target site	Mismatches	CFD score	Indel %	R2	Conclusion
WT (496)	gRNA2					
496-1	gRNA2	2	0.1875	0	0.97	No detectable off-target editing
496-2	gRNA2	2	0.1875	0	0.98	No detectable off-target editing
4496-3	gRNA2	2	0.1875	0	0.97	No detectable off-target editing
WT (499)	gRNA2					
499-1	gRNA2	3	0.3579	0	0.99	No detectable off-target editing
499-2	gRNA2	3	0.3579	0	0.99	No detectable off-target editing
499-3	gRNA2	3	0.3579	0	1.0	No detectable off-target editing
WT (486)	gRNA3					
486-1	gRNA3	3	0.1123	0	0.98	No detectable off-target editing

Indel %: Percentage of Cas9-induced insertions and deletions calculated by ICE software. R^2 (model goodness-of-fit) represents the goodness-of-fit between the Sanger sequencing chromatogram and the ICE indel deconvolution model. Indel % = 0 indicates that no CRISPR-induced

insertion-deletion off-target mutations were detected. (496), Chromosome GWHBISF00000496; (499), Chromosome GWHBISF00000499; (486) Chromosome GWHBISF00000486.

2. Sequence of the RUBY reporter cassette.

ATGGATCATGCGACCCTCGCCATGATCCTCGCGATCTGGTTCATCAGCT
TCCACTTCATCAAGCTGCTGTTCTCCCAGCAGACCACCAAGCTGCTTCC
GCCAGGACCAAAGCCGCTTCCGATCATCGGCAACATCCTTGAGGTGGG
CAAGAAGCCGCATCGGTCCTTCGCCAACCTCGCCAAGATTCACGGCCC
ACTCATTTCCCTCAGACTCGGCTCTGTGACCACCATCGTTGTGTCCTCT
GCCGACGTGGCCAAAGAGATGTTCCTCAAGAAGGATCACCCGCTCTCC
AACCGCACGATCCCGAATAGTGTTACAGCCGGCGACCACCACAAGCT
CACCATGTCTTGGCTCCCGGTGTCTCCGAAGTGGCGCAACTTCCGCAA
GATTACCGCCGTGCATCTGCTCTCCCCACAGAGACTCGATGCCTGCCA
GACATTCAGGCACGCCAAGGTGCAGCAGCTCTACGAGTACGTTCAAG
AGTGCGCCCAGAAAGGCCAGGCCGTGGATATTGGCAAGGCCGCCTTT
ACGACCAGCCTCAACCTCCTCAGCAAGCTGTTCTTCAGCGTCGAGCTG
GCGCACCACAAGTCCCATACCAGCCAAGAGTTCAAAGAGCTGATCTG
GAACATCATGGAAGATATAGGCAAGCCGAACTACGCCGACTACTTCCC
GATTCTCGGCTGCGTTGACCCATCTGGCATTAGAAGAAGGCTCGCCTG
CTCCTTCGACAAGCTGATCGCCGTGTTCCAGGGCATCATCTGCGAGAG
ACTCGCCCCAGATTCCTCCACCACAACTACCACCACCACCGACGACGT

GCTCGATGTGCTCCTCCAGCTGTTCAAGCAGAACGAGCTGACGATGGG

CGAGATCAACCACCTCCTCGTGGACATCTTCGACGCCGGCACCGATAC

CACATCCTCCACATTCGAGTGGGTGATGACCGAGCTGATCCGCAATCC

AGAGATGATGGAAAAGGCCCAAGAGGAAATCAAGCAGGTCCTCGGC

AAGGACAAGCAGATCCAAGAGTCCGACATCATCAACCTGCCGTACCT

CCAGGCGATCATCAAAGAGACACTCCGCCTCCATCCGCCGACCGTGTT

CTTGCTCCCAAGAAAGGCCGACACCGATGTCGAGCTGTACGGCTACAT

CGTGCCGAAGGATGCCCAGATCCTCGTGAACCTCTGGGCCATTGGCAG

GGACCCAAACGCCTGGCAGAACGCCGATATTTTCAGCCCAGAGCGCTT

CATCGGCTGCGAGATCGATGTTAAGGGCCGCGATTTCGGCCTCCTTCCA

TTTGGCGCTGGCCGCAGAATTTGCCCAGGCATGAATCTCGCCATCAGG

ATGCTCACCCTCATGCTCGCCACACTCCTCCAGTTCTTCAACTGGAAGC

TCGAAGGCGACATCTCCCCGAAGGACCTCGACATGGACGAGAAGTTC

GGCATTGCGCTCCAAAAGACCAAGCCGCTCAAGCTCATCCCGATTCCG

CGCTACGGTAGCGGAGCTACCAATTTTAGCCTCCTTAAGCAGGCAGGT

GATGTAGAAGAGAACCCCGGGCCTATGAAGATGATGAACGGCGAGGA

CGCCAACGACCAGATGATCAAAGAGTCCTTCTTCATCACCCACGGCAA

CCCGATCCTCACCGTCGAGGATACACATCCGCTCAGGCCGTTCTTCGA

GACATGGCGCGAGAAGATTTTCTCCAAGAAGCCGAAGGCCATCCTCAT

CATCTCCGGCCACTGGGAGACAGTGAAGCCAACCGTGAACGCCGTGC

ACATCAACGACACCATCCACGACTTCGACGACTACCCAGCCGCCATGT

ACCAGTTCAAGTACCCAGCTCCAGGCGAGCCAGAGCTTGCGAGAAAG

GTGGAAGAGATCCTCAAGAAGTCCGGGTTCGAGACAGCCGAGACAGA

CCAAAAGAGGGGCCTTGATCACGGCGCCTGGGTTCCACTCATGCTCAT

GTATCCAGAGGCGGACATCCCGGTGTGCCAGCTCTCAGTTCAGCCACA

TCTCGACGGCACCTACCACTACAATCTCGGCAGAGCCCTCGCGCCGCT

CAAGAATGATGGCGTGCTCATTATTGGCTCCGGCAGCGCCACACATCC

ACTCGATGAGACACCGCACTACTTCGATGGTGTTGCCCCTTGGGCCGC

TGCCTTCGATTCTTGGCTTAGGAAGGCCCTCATCAACGGCCGCTTCGAG

GAAGTGAACATCTACGAGAGCAAGGCCCCGAACTGGAAGCTCGCCCA

TCCATTTCCAGAGCACTTCTACCCGCTCCACGTTGTGCTCGGCGCTGCT

GGTGAAAAGTGGAAGGCCGAGCTGATCCACTCCTCCTGGGATCATGGC

ACACTTTGCCACGGCTCCTACAAGTTCACCTCCGCCGGATCCGGAGCA

ACCAACTTTAGCCTGCTCAAGCAAGCAGGAGATGTTGAGGAAAATCCT

GGCCCCATGACCGCCATCAAGATGAACACCAACGGCGAGGGCGAGAC

ACAGCACATCCTCATGATCCCGTTCATGGCGCAGGGCCACCTCAGGCC

ATTTCTCGAACTCGCCATGTTCCTCTACAAGCGCTCCCACGTGATCATC

ACCCTGCTCACAACTCCGCTCAATGCCGGCTTCCTCAGGCACCTCCTTC

ACCACCATTCCTACTCCTCCAGCGGCATCAGGATCGTCGAGCTGCCATT

CAACTCCACCAACCACGGACTCCCACCGGGCATCGAGAACACCGATA

AGCTCACACTCCCGCTCGTGGTGTCCCTCTTCCATTCCACCATCAGCCT

CGATCCGCACCTCCGCGATTACATCTCCAGGCATTTCAGCCCAGCCAG

GCCACCACTCTGCGTGATCCATGATGTGTTCCTCGGCTGGGTTGACCAG

GTGGCCAAGGATGTGGGCTCTACAGGCGTGGTGTTCACAACAGGCGG

CGCTTATGGCACATCCGCCTACGTGTCCATCTGGAACGATCTCCCGCAC

CAGAACTACTCCGACGACCAAGAGTTCCCGCTGCCAGGCTTCCCAGA

GAACCATAAGTTCCGCAGGTCCCAGCTCCATCGGTTCCTCAGATATGCC

GACGGCTCCGACGATTGGTCCAAGTATTTCCAGCCGCAGCTCCGCCAG

TCCATGAAGTCTTTTGGCTGGCTCTGCAACTCCGTGGAAGAGATCGAG

ACACTCGGCTTCTCCATCCTCCGCAACTACACCAAGCTGCCGATCTGG

GGCATCGGCCCACTTATTGCTTCCCCAGTGCAGCACTCCTCCTCCGACA

ACAATTCAACAGGCGCCGAGTTCGTGCAGTGGCTCAGCCTCAAAGAG

CCGGACTCCGTCCTCTACATCTCCTTCGGCTCCCAGAACACGATCAGC

CCGACGCAGATGATGGAACTCGCTGCTGGCCTTGAGTCCTCCGAGAAG

CCATTCCTCTGGGTGATCAGAGCCCCGTTCGGCTTCGACATCAACGAA

GAGATGCGCCCAGAGTGGCTGCCAGAGGGCTTTGAGGAACGCATGAA

GGTGAAGAAACAGGGCAAGCTCGTGTACAAGCTCGGCCCGCAGCTTG

AGATCCTCAACCATGAATCCATCGGCGGCTTTCTCACCCACTGCGGAT

GGAACAGCATCCTTGAGTCTCTTCGCGAGGGCGTTCCGATGCTTGGAT

GGCCACTTGCTGCCGAGCAGGCCTACAACCTCAAGTACCTCGAAGATG

AGATGGGCGTCGCGGTTGAGCTTGCTAGAGGCCTCGAAGGCGAGATCT

CCAAAGAGAAGGTCAAGCGCATCGTCGAGATGATCCTTGAGCGCAAC

GAGGGCTCCAAAGGCTGGGAGATGAAGAATCGCGCCGTGGAAATGGG

CAAAAAGCTCAAGGACGCCGTGAACGAGGAAAAAGAGCTGAAGGGC

TCCTCCGTGAAGGCGATCGACGATTTCCTCGACGCCGTCATGCAGGCC

AAACTTGAGCCAAGCCTCCAGTGATAGTGAATATGAAGATGAAGATGA

AATATTTGGTGTGTCAAATAAAAAGGTTGTGTGCTTAAGTTTGTGTTTTT

TTCTTGGCTTGTTGTGTTATGAATTTGTGGCTTTTTCTAATATTAAATGAA

TGTAACATCTCATTATAATGAATAAACAAATGTTTCTATAATCCATTGTG

AATGTTTTGTTGGATCTCTTCTCCAGCATATAACTACTGTATGTGCTATG

GTATGGACTATGGAATATGATTAAAGATAAG

CYP76AD1, P2A, DODA, Glucosyl transferase, HSP18.2

2. Sequence of the *pGTR* vector.

GGGCGAATTGGGCCCGACGTCGCATGCTCCCGGCCGCCATGGCGGCCG

CGGGAATTCGATTCTGCCATGCACCAGCCGGGAATCGAACCCGGGTCT

GTACCGTGGCAGGGTACTATTCTACCACTAGACCACTGGTGCTTTGTTG

CACCGACTCGGTGCCACTTTTTCAAGTTGATAACGGACTAGCCTTATTT

TAACTTGCTATTTCTAGCTCTAAAACCGAGACCTCGGTCTCCAATCACT

AGTGAATTCGCGGCCGCCTGCAGGTCGACCATATGGGAGAGCTCCCAA

CGCGTTGGATGCATAGCTTGAGTATTCTATAGTGTCACCTAAATAGCTTG

GCGTAATCATGGTCATAGCTGTTTCCTGTGTGAAATTGTTATCCGCTCAC

AATTCCACACAACATACGAGCCGGAAGCATAAAGTGTAAAGCCTGGG

GTGCCTAATGAGTGAGCTAACTCACATTAATTGCGTTGCGCTCACTGCC

CGCTTTCCAGTCGGGAAACCTGTCGTGCCAGCTGCATTAATGAATCGG

CCAACGCGCGGGGAGAGGCGGTTTGCGTATTGGGCGCTCTTCCGCTTC

CTCGCTCACTGACTCGCTGCGCTCGGTCGTTCGGCTGCGGCGAGCGGT

ATCAGCTCACTCAAAGGCGGTAATACGGTTATCCACAGAATCAGGGGA

TAACGCAGGAAAGAACATGTGAGCAAAAGGCCAGCAAAAGGCCAGG

AACCGTAAAAAGGCCGCGTTGCTGGCGTTTTTCCATAGGCTCCGCCCC

CCTGACGAGCATCACAAAAATCGACGCTCAAGTCAGAGGTGGCGAAA

CCCGACAGGACTATAAAGATACCAGGCGTTTCCCCCTGGAAGCTCCCT

CGTGCGCTCTCCTGTTCCGACCCTGCCGCTTACCGGATACCTGTCCGCC

TTTCTCCCTTCGGGAAGCGTGGCGCTTTCTCATAGCTCACGCTGTAGGT

ATCTCAGTTCGGTGTAGGTCGTTCGCTCCAAGCTGGGCTGTGTGCACGA

ACCCCCCGTTCAGCCCGACCGCTGCGCCTTATCCGGTAACTATCGTCTT

GAGTCCAACCCGGTAAGACACGACTTATCGCCACTGGCAGCAGCCAC

TGGTAACAGGATTAGCAGAGCGAGGTATGTAGGCGGTGCTACAGAGTT

CTTGAAGTGGTGGCCTAACTACGGCTACACTAGAAGAACAGTATTTGG

TATCTGCGCTCTGCTGAAGCCAGTTACCTTCGGAAAAAGAGTTGGTAG

CTCTTGATCCGGCAAACAAACCACCGCTGGTAGCGGTGGTTTTTTTGTT

TGCAAGCAGCAGATTACGCGCAGAAAAAAAGGATCTCAAGAAGATCC

TTTGATCTTTTCTACGGGGTCTGACGCTCAGTGGAACGAAAACTCACGT

TAAGGGATTTTGGTCATGAGATTATCAAAAAGGATCTTCACCTAGATCC

TTTTAAATTAAAAATGAAGTTTTAAATCAATCTAAAGTATATATGAGTAA

ACTTGGTCTGACAGTTACCAATGCTTAATCAGTGAGGCACCTATCTCAG

CGATCTGTCTATTTCGTTCATCCATAGTTGCCTGACTCCCCGTCGTGTAG

ATAACTACGATACGGGAGGGCTTACCATCTGGCCCCAGTGCTGCAATG

ATACCGCGAGACCCACGCTCACCGGCTCCAGATTTATCAGCAATAAAC

CAGCCAGCCGGAAGGGCCGAGCGCAGAAGTGGTCCTGCAACTTTATC

CGCCTCCATCCAGTCTATTAATTGTTGCCGGGAAGCTAGAGTAAGTAGT

TCGCCAGTTAATAGTTTGCGCAACGTTGTTGCCATTGCTACAGGCATCG

TGGTGTCACGCTCGTCGTTTGGTATGGCTTCATTCAGCTCCGGTTCCCA

ACGATCAAGGCGAGTTACATGATCCCCCATGTTGTGCAAAAAAGCGGT

TAGCTCCTTCGGTCCTCCGATCGTTGTCAGAAGTAAGTTGGCCGCAGTG

TTATCACTCATGGTTATGGCAGCACTGCATAATTCTCTTACTGTCATGCC

ATCCGTAAGATGCTTTTCTGTGACTGGTGAGTACTCAACCAAGTCATTC

TGAGAATAGTGTATGCGGCGACCGAGTTGCTCTTGCCCGGCGTCAATA

CGGGATAATACCGCGCCACATAGCAGAACTTTAAAAGTGCTCATCATT

GGAAAACGTTCTTCGGGGCGAAAACTCTCAAGGATCTTACCGCTGTTG

AGATCCAGTTCGATGTAACCCACTCGTGCACCCAACTGATCTTCAGCAT

CTTTTACTTTCACCAGCGTTTCTGGGTGAGCAAAAACAGGAAGGCAAA

ATGCCGCAAAAAAGGGAATAAGGGCGACACGGAAATGTTGAATACTC

ATACTCTTCCTTTTTCAATATTATTGAAGCATTTATCAGGGTTATTGTCTC

ATGAGCGGATACATATTTGAATGTATTTAGAAAAATAAACAAATAGGG

GTTCCGCGCACATTTCCCCGAAAAGTGCCACCTGATGCGGTGTGAAAT

ACCGCACAGATGCGTAAGGAGAAAATACCGCATCAGGAAATTGTAAG

CGTTAATATTTTGTTAAAATTCGCGTTAAATTTTTGTTAAATCAGCTCATT

TTTTAACCAATAGGCCGAAATCGGCAAAATCCCTTATAAATCAAAAGA

ATAGACCGAGATAGGGTTGAGTGTTGTTCCAGTTTGGAACAAGAGTCC

ACTATTAAAGAACGTGGACTCCAACGTCAAAGGGCGAAAAACCGTCT

ATCAGGGCGATGGCCCACTACGTGAACCATCACCCTAATCAAGTTTTTT

GGGGTCGAGGTGCCGTAAAGCACTAAATCGGAACCCTAAAGGGAGCC

CCCGATTTAGAGCTTGACGGGGAAAGCCGGCGAACGTGGCGAGAAAG

GAAGGGAAGAAAGCGAAAGGAGCGGGCGCTAGGGCGCTGGCAAGTG

TAGCGGTCACGCTGCGCGTAACCACCACACCCGCCGCGCTTAATGCGC

CGCTACAGGGCGCGTCCATTCGCCATTCAGGCTGCGCAACTGTTGGGA

AGGGCGATCGGTGCGGGCCTCTTCGCTATTACGCCAGCTGGCGAAAGG

GGGATGTGCTGCAAGGCGATTAAGTTGGGTAACGCCAGGGTTTTCCCA

GTCACGACGTTGTAAAACGACGGCCAGTGAATTGTAATACGACTCACT

ATA

3. Sequence of *EuSQS*

ATGGGGAGTTTGGGGACGCTGCTGAAGCATCCGGATGATTTGTATCC

GCTGTTGAAGCTGAAGAGGGCGGTGAAGCATGCGGAGAAGCAGATCC

CTCCGGAGCCGCACTGGGCATTCTGTTACACCATGCTCCACAAGGTCT

CCCGTAGTTTCGGA**CTCGTCATTCAACAGCTCGGAACGGAGC**TTCGC

AACGCTGTAAGTTGATGTTAATCGCATTTCTATGGATATCTCTTCATCAC

TCCAACGAATTTGTGAATATTTTTTTTTGTACGCCTGCTCATTTGCATGC

GTGCACAAATGCATCTGTATTGATGTATTTGTGGCGTGTCTGTCAATTCT

ACGGTTATGAAATTGACAAAAGCAATAATCAAGCCTCCGGCTGTATTA

AGTCACATGGATCGGTCTGTGTCGGTAATAATTGTTTGTTTCTTCTGTAT

ACTGTTAGCGTTATTGTTGATGATGGTGGTATAATCGCTGAAAAATATAT

CACTATGAGTTCACATGGAGGTACTTCAGTATAGAAAATTATCCATACA

AAAGCCATTTAAATCCCTCTGCATATTCATTTTGTACTTCATAATTTGATG

AACCTTCGTGTGGATGAAATGAAAGTGATACAGACGAAGTTACACATT

CAGTTCTTTTCCAATTTGTGTACTCTCATTACCTTTTCAGTGGCGGCAGA

TCTTGGATTTTGGAGTGGGGGGTGAATGATACTGATTCATACATGTTTGA

TTCCTATCCCAAACTACTATATAAATCAAATGAGTTACTCCACTTTGGCA

CCATATGGTTTTAATTGGAACCTCCTAACATGGTGTCAAAGCAAAGGTT

ATGAATCTCTCGTTCCCTCGTATACAAATCATTTGTGCAACCTGGGGTC

GTACATTTCTCATCATTCCCTCGTAATCGAATCATCTGTGCAAACCGGG

GGTTACACTTAAGGGCGGGTGTGAGTGTTAGATTCTTGTCCCATATCGA

TCACTTGAGAGTTTCCAAAGTACTATATAAATCAAATGAGCTACTCCAT

TTGGCACCATATAGTTTTAAGTTGGAACCTCATAACAATACACAAGGGC

TATTGTACTGAAAGTGTTTTACTTTTCTCTCTCAAGATCCTTCAGATTCC

ATCATTTGCCTTTGCCATCTCTGATCCACAATCTAGCATTTCCTTTAACA

CAGGCACTTACTTAAACATGGCAATTTCTAGAGGAAAAGGGATTAAAT

CCATGACTATGCACCGTGTTGTGTCAGTGCCTTGTGCCGTTCCACCTTA

GAGAACTCAACATCACAGCATTAGTCCCACATAGATGTTCCTGCCTACT

AAATTACTTATCCAAAAAAAATAAAAAGAAAAAGAAAAAAAGAAAA

AGAAAAAAGTAATTTACAGGCAAGCCAAAAAGAAAAGAAAAGAAAA

GAAAATGAAAGAGAGTCTTTTTTGTTGAGTTCTGATTAGTCATGCACTT

ATTTTTCCCATTTTGCAGGTATGCATCTTTTATTTGGTTCTTCGAGCCCTT

GACACTGTTGGTATGTTCTATTATCTTTAAGCTCTTGTTTTCTCTATGCCA

TTTCAATTTATGCCTAATTATACTGTGATCGGCCTGTTGTGTTATTTTCTG

CATTAATTTTGCCACTCTCTCTTGAATTTGACGAAATTTATTCTCACACA

TATATTTGAATTTTTTGCCATGTTAGTTTGTACATCACCTCCCATCTCATT

TACTTTTGTGTTTTCCCTTTTGCCAGAGGATGATACGAGCATATCCACTG

AAGTGAAAGTACCGATACTGATGGCTTTTCATCGCCACATATATGATCG

TGAATGGCACTTTTCATGTGAGTTCCATCCCCCCTCCCCACATGGCTCC

TACCACACTTTTGAAACCTGCGTTTACCACACTGCCTAGAGAGAGAGA

GAGATGAATATTGGTATACTACTTTTATAGATGCAGGTGTTAAAAACAA

ATCCATTTTCCATTTTCCTATATTCATTTCCTTTTCACTTCTTCGCCAGAA

CTTTTAGTTGGCCAGGGGTTTGAATTTGTTACTTCTCCATCACGTTTTTC

CTATTTTTACATGTAGGATGGTAAGGCCCCGTTTGTTTACTGTTTCCGTTT

CTTGATTTTTGTTCCCGCTTCCAAAATTTTGGGGTTAATCTATGGAAATG

CGTTCGGTAAACCCATTTCTATTCCAAAATTTTGGAAATAATTTGGGAG

TAATTCCTGAATTCCCTAAGAATTTGAGAAACAAAAAAATACTCATTCC

TATTCACTCCCAAGAAACTATTCCCACTATCACCTATTGGCCACCACCA

CCCTGCTGGCCACTATAAATTTAAAGGAAAAAAGGAACAAATGTTGC

AACCCAAGCGTGTTTCTTGTTTCTGTTCTCATAAATTAAAAGAAACAAT

TTCAGGAAAAATGGTTGTTACCAAACATGGTTTTATTCCCAGAATACTG

CTGAACGGAAAAAAATTCTTATCCCAATTTATTGGATGAGAATTAGAGT

TTGTTTTGTTTGTCAGCCGGCAGCCCTTCAATACCTGATAGGATACCAAT

ATTTTATTTTATTATTTTTTTTTTTCAAATTGTGGATTTTATTGCTAATTTTT

GTTCTTGCCTCTTTTGTAAGGTGGTACAAAGGAATACAAAGTTCTCATG

GATCAGTT**CCATCATGTTTCAACCGCTTTTCTGGAGC**TTGGAAGCAG

GTTAGCGACTTTGAAATTTTTCTCTCCATATGTGCATATATATATCGATGT

TGTAAGAGAAACTTACACACAATGTTCTGCAGTTATCAGGAGGCAATT

GAAGATATTACCGAGAGAATGGGCGCAGGCATGGCAAAATTTATATGC

AAGGAGGTATGAACGTAGTATATGCACGAGAGGATGCTTCATACATTTT

CCGATTTTTTTGTGAATTCGAAAAAAACTAATTGCTTTTTGAATATATAT

ACATCTATGGGGTCCCGGGTTCAAATTTTGAAAATAGTCTCCCTATATAC

AGGGATAAGGTTGATTACATCAGACCCTTCCTAAACTCCGCAATGGTA

GGAGTTTTCGTGCACTGGGTTGTTCTTTATATATATATATATATTTTATTTT

ATTTTTTAATTTCTTCGATTTTTATATTTAAAGAGATTGATAGACTAGGAT

ATGACCCCTCTCCACCTAGATTTTCTGAACCCTCTACGGAATTCTTTTG

GATTAAGAGATCGTATAACACTAAGATATCATCTCTACTTTGTCAAACAT

ATTTTTTATTTGTTTATGGTGTTTTTAAGTAAAAATACTTTTTATTCAATTT

TTAATTATAGTAAGTAGTTTTAAAAAAATGCTACCAAACAGAGCCTTAC

TCATACACGTTTTCTATCTTCTTGGCAGGTGGAAACAATTGACGACTAT

GACGAGTATTGTCACTATGTGGCCGGACTTGTTGGATTAGGATTGTCA

AAGCTTTTTCATGCTGCCGGGACTGAAAAATTGTTTTCCGATTCCATCT

CCAATTCAATGGGTTTATTTCTTCAGGTAATTTCTTTTCCAATGCCGCCC

TAAAAAGTGACATTTTCCATTTTCAATCTTCCCTTGTGTTTAGAAATACG

ATTTTCATTTATTTATTTTTTGCGTTATTAATTGCAGAAAACGAACATTAT

TCGAGATTATCTGGAGGACATAAACGAGATCCCAAAATCGCGGATGTT

TTGGCCTCGCCAAATTTGGAGTAAATATGTCAACAAACTCGAGGTTCTT

CTCTTACACCTACTTGATCAAATTTTTAAAACTAAGCCTTAGTCTCACTT
ATGTAGGGTTGGCTACTTGAATCCATTGATGAAATTTTTAAAAGACGAG
TGAAAAAGAA**TGAGAAGGCTTAGGCCCTGTTTGG**TTGAGAATTTGG
ATACTGTTTTCACTTTTCAAAAACTCAAAATATATATATATTTTTTTTTTG
TTTTTGACAACAGAACTCAAAAAGGCATTTACAAACAAGTGTCACAAT
TTTCAAAATTTAATTTCAACTTTTTTTTACATCTTTTTTTTTATATATATATT
TTTCTACCACATATTCACTTAGCAAAAATCATTGTTCTCTCTCTTTTTTAA
CATCTATCTCAATCAATCATATCACTTTTTACTTATCAAAATTTTAACACA
TTATTAATAAGATATTCTCTTTTTCACAACTTTTTCAAATCTGAACCAAA
CAACTTTCACAAATTGTTCTCCAAATTTGTTTTCAAAATTTTCGGAAACT
ATTTTCGAAAACTAAACCAAACATGCCCTCAACTTCTCTGTCAACCAA
ATAGGAAAACAACACCGAAACCCTAAATATAAGAGAATGGTAGAAAT
GCCCTCTAACACATCTTGTAATGCATTGTGAATCAATGTTGGGTACTTTT
TGCTGTATTGCTTCAGGACTTAAAAGACGAGGAGAACTCGGAGAAGG
CAGTGCAGTGTCTGAACGATATGGTCACAAATGCTTTGATACATGTAGA
AGATTGTCTGATATACATGTCAGAATTACGTGATCCTGCTATCTTCCGGT
TTTGTGCTATTCCACAGGTACATAAATGCTTTTCAAAATGGTTTATTTTC
CGCTTAGCACATTGTTTTACTTAGCGCTCTCATGCACACACACACATATA
TGTATGTTGAATTGCTCGGGTTACCTGATCCTGAGCTATATATAAATCTA
GAAGCAATCCTCATCTTACAAGACGTCTGTTGAGAGTAAGTCAAGCCT
CATTGGGTTTCTACAATATATATATACATACATATGTGTGTGTGTATTTGG
CTCCTATTTTGAATTGTATCCTTCATGAGGGTGTTATGTTCATATGAAAG

TGAAAATTTGTCTGTGATGTTTGGTGTTTTTACCAGATCATGGCGATTGG

GACGCTAGCTTTATGCTACAACAATATTGAAGTCTTCAGGGGGGTCGTA

AAAATGAGACGCGGTAAAATAACTTTCTGTTCATAAAAATACTTGGGC

ACTATTTGATAACGTTTTTTTTTTTTTTTTTTTTTAAATATTGTTTGTTTTGC

ATATATTTGGGCATTCTTCTAATGATTTTAGGGGTTTGTTTTGATTAGGTC

TGACGGCAAAAGTAATTGATAGGACAAGAACAATCTCGGATGTGTATG

GTGGTTTCTTTGATTTTTCTTGCATGCTCAAGTCCAAGGTAATCTGCTTC

TTCATGTTTTTCCACACTACCCTAACAACACTCACTCTTCAACAATTTTT

TTTTTTTTCTAAAATTATTGGATGCAATTAAATATACAATGCAAATGGTA

AAAATTTGATTAAATCATAGACAAATGGAGAAATAATTGAAAAAAAAC

TATACCTGAAAATTTAACTACACTAAATGAATTGTATTTTTTATTTTTTTT

TTGTAAATTTTATTAAATTATAAGTAAACGAAGAAATATATAAATTAATT

TATTGAATATAAGATAAACTATTAATAAATTAGGATGTAATGATATTTTA

ACAGTCAAGATTTAAGTTTAAAAGATGAATGGTTGAGATTGAATTTGTG

CAATCACTCAATCTAGTAACTTCTAACTTCTCTACCCTATTCACTCCACT

CAACCCACTCAATAGTTTAATTATACTTTGAACCCTTTAAGTTATATATAT

ATATATATATATATATATATATATATATATATATATATTATTAACTTCATGTAA

TATAGTGCCTTTAGGCATGGAGAATCCATAGTCTATTAAGAAATCATAA

CGGAAAGATATTTTAGCGGTCCAGATGAATGGTTGAGATTGAATTTCTA

CAATTTCTCAACACTGATGTCAAATTTATCTACATTACTCAACCGACTCT

ATAAATTAGATGAAGAGATGAAATATTTGGAGGAAAAATTCTCGCTTA

AAAATTTCACGTTTTTACTTTATACTCTTCCAAGTTTCATATCTGTACTTT

ATTAACTGTTTTTTAACACTTACAAATTTAAAAATCGATATAGTGTCTTT
AGGCTAATTCTACTTCTAACACATTCCCCCTCCCCCTCCCCTTTTTTCGA
CTATTTATTTGCCATTATAGGTGGATAAAAATGATCCGAATGCTACAAA
AACATTGAACCGGCTGGATGCAATTCAGAAAGTCTGCAGAGAATCCG
GCACCTTGACCGGAAGGTATATGCTCAAACTTTCTTCTCTTGGGTAGTG
TTTGGCTCCGAAATGGAATTAGAAATAAACGCATGGAAATTTTAATTCC
AATTCCATGAAACCGAACAGACCTTTTAATGGATTTCAAAATTCTGCGT
GTAAACACTTGTTTATCCGAACAATGAAATTATTCATTTAAAATAGTTAT
CCAAACAAATACATAATTGATGGGATTTAAAAATTTAGAATTTTAAAAT
CTCATCATTTTCAAAAACGCATAATTCAAAAACACATTTACAAAATAAT
TTTCATAATATTCAAATTATCACAATTACTTGCTTTTAATGTTATATTTTTA
TAATTTTATAAATTTAATTTTAGGTTTAATTCTAAATCTTTATTTTTTATTG
GTCTGTAAATTTTTTATACCGCACATTCACCTAACAAACATCATTGTTCT
CTTTTTGCAGGAAATCTTACATTCTGAGTGGTGAACCAAGATATAATTC
TGTTCTGGTGAGTTTTTTTCTTTCTTTTTTTCTTTTTTATTTTTATCCTTATT
TTAAAATAAAAATTAAAAATTAAAAAAAATGTGCTTTAGTTTTGATGTA
ATTATTGAATGAATGTAGGTGGTGGTGGTCTTCATCATACTGGCTATCCT
TCTGGCTTATCTATCTGCCAACAGAGCAAGCAATCTGTGTAA

4. The sequence of PTG (*EuSQS*), tRNA-gRNA1-tRNA-gRNA2-tRNA-gRNA3

AACAAAGCACCAGTGGTCTAGTGGTAGAATAGTACCCTGCCACGGTAC

AGACCCGGGTTCGATTCCCGGCTGGTGCA**GGACTCGTCATTCAACAG**

CTGTTTTAGAGCTAGAAATAGCAAGTTAAAATAAGGCTAGTCCGTTATC

AACTTGAAAAAGTGGCACCGAGTCGGTGCAACAAAGCACCAGTGGTC

TAGTGGTAGAATAGTACCCTGCCACGGTACAGACCCGGGTTCGATTCC

CGGCTGGTGCA**GATTCCATCTCCAATTCAAT**GTTTTAGAGCTAGAAAT

AGCAAGTTAAAATAAGGCTAGTCCGTTATCAACTTGAAAAAGTGGCAC

CGAGTCGGTGCAACAAAGCACCAGTGGTCTAGTGGTAGAATAGTACCC

TGCCACGGTACAGACCCGGGTTCGATTCCCGGCTGGTGCATAAACGAG

ATCCCAAAATCGGTTTTAGAGCTAGAAATAGCAAGTTAAAATAAGGCT

AGTCCGTTATCAACTTGAAAAAGTGGCACCGAGTCGGTGC

tRNA, **gRNA1**, **gRNA2**, **gRNA3**, and gRNA scaffold

5. Construction of the PTG component by Golden Gate assembly.

5.1 DNA fragment amplification

Regent	Volume
pGTR plasmid	0.1 ng
5 x PrimeSTAR Buffer	10 µl
dNTP	4 µl
Forward primer (10 µM)	2.5 µl
Reverse Primer (10 µM)	2.5 µl
PrimeSTAR DNA Polymerase (1.25 U/µl, Takara)	0.5 µl
H_2O	x µl
Total	50 µl

5.2 Fragment amplification primer set

PCR ID	Forward primer	Reverse Primer	parts symbol (L1)
P1	L5AD5-F	gRNA1-R	L5AD-gRNA1
P2	gRNA1-F	gRNA2-R	gRNA1-gRNA2
P3	gRNA2-F	gRNA3-R	gRNA2-gRNA3
P4	gRNA3-F	L3AD5-R	gRNA3- L3AD5-R

5.3 PCR was run with the following program:

Temperature	Time	cycles
98 ℃	2 min	1
98 ℃	30 s	35
58 ℃	20 s	
72 ℃	20 s	
72 ℃	2 min	1
4 ℃	Hold	1

5.4 The PCR products were purified using a gel extraction kit (Tiangen).

5.5 Individual parts were ligated together by GG assembly:

L1 parts	50 ng (add equal amount for each part)
2 x T7 DNA ligase Buffer (NEB)	10 μl
Bovine Serum Albumin (1mg/ml)	2 μl
Bsa I (10 U/μl, NEB)	0.5 μl
T7 DNA Ligase (3000 U/μl, NEB)	0.5 μl

Total	20 μl

5.6 Reaction conditions of GG:

Temperature	Time	cycles
37 ℃	5 min	1
20 ℃	10 min	50
20 ℃	60 min	1

5.7 The GG reaction product was diluted 10-fold.

5.8 Assembly fragments P1, P2, P3, and P4.

Regent	Volume
GG reaction product	0.1 ng
5 x PrimeSTAR Buffer	10 μl
dNTP	4 μl
S5AD5-F (10 μM)	2.5 μl
S3AD5-R (10 μM)	2.5 μl
PrimeSTAR DNA Polymerase (1.25 U/μl, Takara)	0.5 μl
H_2O	x μl
Total	50 μl

5.9 PCR was run with the following program:

Temperature	Time	cycles
98 ℃	2 min	1
98 ℃	30 s	35
58 ℃	20 s	
72 ℃	60s	

72 ℃	2 min	1
4 ℃	Hold	1
